# Scalable Fabrication of Reversible Antifouling Block Copolymer Coatings via Adsorption Strategies

**DOI:** 10.1021/acsami.3c01060

**Published:** 2023-04-05

**Authors:** Anna M. C. Maan, Chantal N. Graafsma, Anton H. Hofman, Théophile Pelras, Wiebe M. de Vos, Marleen Kamperman

**Affiliations:** † Polymer Science, Zernike Institute for Advanced Materials, University of Groningen, Nijenborgh 4, 9747 AG Groningen, The Netherlands; ‡ Macromolecular Chemistry and New Polymeric Materials, Zernike Institute for Advanced Materials, University of Groningen, Nijenborgh 4, 9747 AG Groningen, The Netherlands; § Membrane Science and Technology, MESA+ Institute for Nanotechnology, University of Twente, P.O. Box 217, 7500 AE Enschede, The Netherlands

**Keywords:** antifouling coating, two-step adsorption, polymer brush, diblock copolymers, complex coacervate core micelles, hydrophobic surfaces

## Abstract

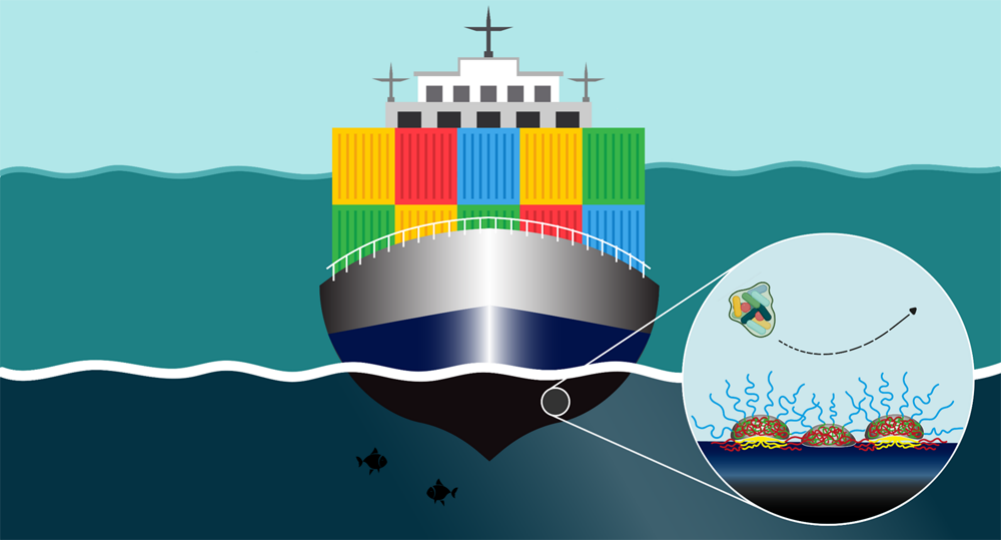

Fouling remains a widespread challenge as its nonspecific and uncontrollable character limits the performance of materials and devices in numerous applications. Although many promising antifouling coatings have been developed to reduce or even prevent this undesirable adhesion process, most of them suffer from serious limitations, specifically in scalability. Whereas scalability can be particularly problematic for covalently bound antifouling polymer coatings, replacement by physisorbed systems remains complicated as it often results in less effective, low-density films. In this work, we introduce a two-step adsorption strategy to fabricate high-density block copolymer-based antifouling coatings on hydrophobic surfaces, which exhibit superior properties compared to one-step adsorbed coatings. The obtained hybrid coating manages to effectively suppress the attachment of both lysozyme and bovine serum albumin, which can be explained by its dense and homogeneous surface structure as well as the desired polymer conformation. In addition, the intrinsic reversibility of the adhered complex coacervate core micelles allows for the successful triggered release and regeneration of the hybrid coating, resulting in full recovery of its antifouling properties. The simplicity and reversibility make this a unique and promising antifouling strategy for large-scale underwater applications.

## 1 Introduction

Transport vessels, water purification devices, and sewage systems are designed to excel in aqueous environments, which they do remarkably well until nonspecific adhesion of materials from the surrounding environment occurs. This undesirable and uncontrollable fouling process substantially limits the performance of these systems and numerous other applications.^1−4^ Many types of fouling exist, including organic, inorganic, composite, and biological fouling with the latter being the most prominent in underwater systems.^5^ Biological fouling (i.e., biofouling) is defined by the settlement and accumulation of unwanted biological matter on surfaces, which ultimately leads to the formation of biofilms and macroscopic biofouling.^6−8^ Within marine environments, this aquatic growth appears on transport vessels, giving rise to increased frictional drag, fuel consumption, chemical waste and harmful gas emissions, and dispersal of invasive marine species.^6,7,9,10^ Complications of fouling also extend to industrial applications (pipe blockage, decreased membrane flux, and water contamination) and biomedical applications (increased risk of infection, implant rejection, and biosensor failure).^6,11−14^

Due to the significant environmental and economic impact of (bio)fouling, many types of protective coatings have been developed to reduce or even prevent fouling, including antimicrobial coatings (metals and enzymes),^15,16^ natural and bio-inspired coatings,^6,13,17^ and polymer-based coatings.^18,19^ The latter proved to be highly promising as they showed superior characteristics compared to conventional coatings: they are affordable, non-toxic, biocompatible, easy to process, and have a wide-range efficacy, and their functionalities are easily modified to suit the application of interest.^3^ The class of polymer-based antifouling coatings is mostly dominated by a large variety of polymer brushes, which can be defined as densely end-grafted arrays of polymer chains. These brushes can act as excellent barriers to keep fouling agents away from the surface on the grounds of both entropic (steric repulsion) and enthalpic (tightly bound hydration layer) contributions. The degree of steric repulsion generated by the polymer brush is strongly determined by the density of the brush.^20−27^

However, most of these polymer-based coatings suffer from serious limitations, particularly in their scalability, which has hampered their implementation. The majority of these polymer brush coatings are generated via well-known and controllable covalent methods, including grafting to or grafting from techniques. These covalent anchoring strategies can produce stable and high-density brushes, leading to effective antifouling coatings, but the reaction protocols are often complicated and too expensive to extend beyond the lab scale. Moreover, fouling will inevitably occur, and the removal of an irreversibly bound coating is expensive and requires harsh cleaning agents. In order to render the surface antifouling again, it has to be recoated completely, which makes it a costly and environmentally unfriendly strategy.^28−34^ Finally, these protective layers are typically optimized for charged and/or hydrophilic substrates, which hinders their use in many other applications that involve hydrophobic surfaces.^25,26,35^

The challenges of covalent anchoring strategies may be overcome by shifting focus to different coating methods, such as adsorption-based protocols. Physisorption (i.e., physical adsorption) relies on van der Waals and hydrophobic interactions.^28^ Fabricating coatings via physisorption can offer many advantages, including simplicity, cost-effectiveness, large-scale applicability (e.g., via spray-painting or dip-coating), and eco-friendly renewability.^3,29,31−34^ Many adsorption strategies already exist that can produce highly effective antifouling coatings on charged and/or hydrophilic surfaces, such as physisorbed PLL-*g*-PEG^36−39^ or PMPC brushes^40^ and chemisorbed PDMS brushes,^41^ but these methods do not easily extend to hydrophobic surfaces. In fact, developing high-density brushes directly applicable to hydrophobic substrates is considered more strenuous as these surfaces are less prone to chemical interactions and surface modifications are no longer straightforward. Still, they are essential for many (large-scale) surfaces found in a range of applications (e.g., piping, tubing, packaging, and containers), and it is therefore a central component in this work.^42^ An interesting example of a physisorbed system that is applicable to hydrophobic surfaces involves the one-step adsorption of complex coacervate core micelles (C3Ms), also known as polyion complex (PIC), block ionomer complex (BIC), or interpolyelectrolyte complex (IPEC) micelles.^43,44^ C3Ms are formed by the electrostatic attraction between two oppositely charged polyelectrolytes of which at least one is connected to a neutral and water-soluble block. The core of the self-assembled C3M comprises complexed polyelectrolytes, while the neutral chains form the corona.^27,43,45−47^ Several studies have shown that these C3Ms can function as antifouling agents once adsorbed to either (charged) hydrophilic or hydrophobic surfaces.^27,43,47,48^ The liquid-like core of the C3Ms would facilitate spreading onto the surface and subsequent rearrangement into a brush layer, while traditional polymeric micelles with a hydrophobic core tend to adsorb intactly.^27^ In addition, since C3Ms are responsive to changes in pH and salt concentrations, this allows for an easy removal and renewal of the coating once fouled.^47^ However, the density and homogeneity of C3M coatings were found to be rather low and insufficient to fully suppress the adsorption of proteins, especially when fabricated on hydrophobic surfaces.^27,47^

Another promising physisorption approach was developed by de Vos et al.^24,49^ Following a two-step adsorption procedure called the zipper brush method, a diblock copolymer is complexed to a pre-adsorbed polyelectrolyte brush. By tuning the density and degree of polymerization of the polyelectrolyte brush as well as the degree of polymerization of the charged block of the diblock copolymer, a grafting density comparable to or even higher than covalent polymer brushes can be obtained. As a result, they managed to produce stable and ultradense polymer brushes via adsorption, even on hydrophobic surfaces. Unfortunately, the formation of these “zipper brushes” required the use of a time-consuming Langmuir–Blodgett technique, which limited its translation to large-scale applications.^24,49^ Hence, until now, most physisorbed systems suffer from either a low and uncontrolled surface density or time-consuming protocols.^20,22,28^

**Figure 1:**
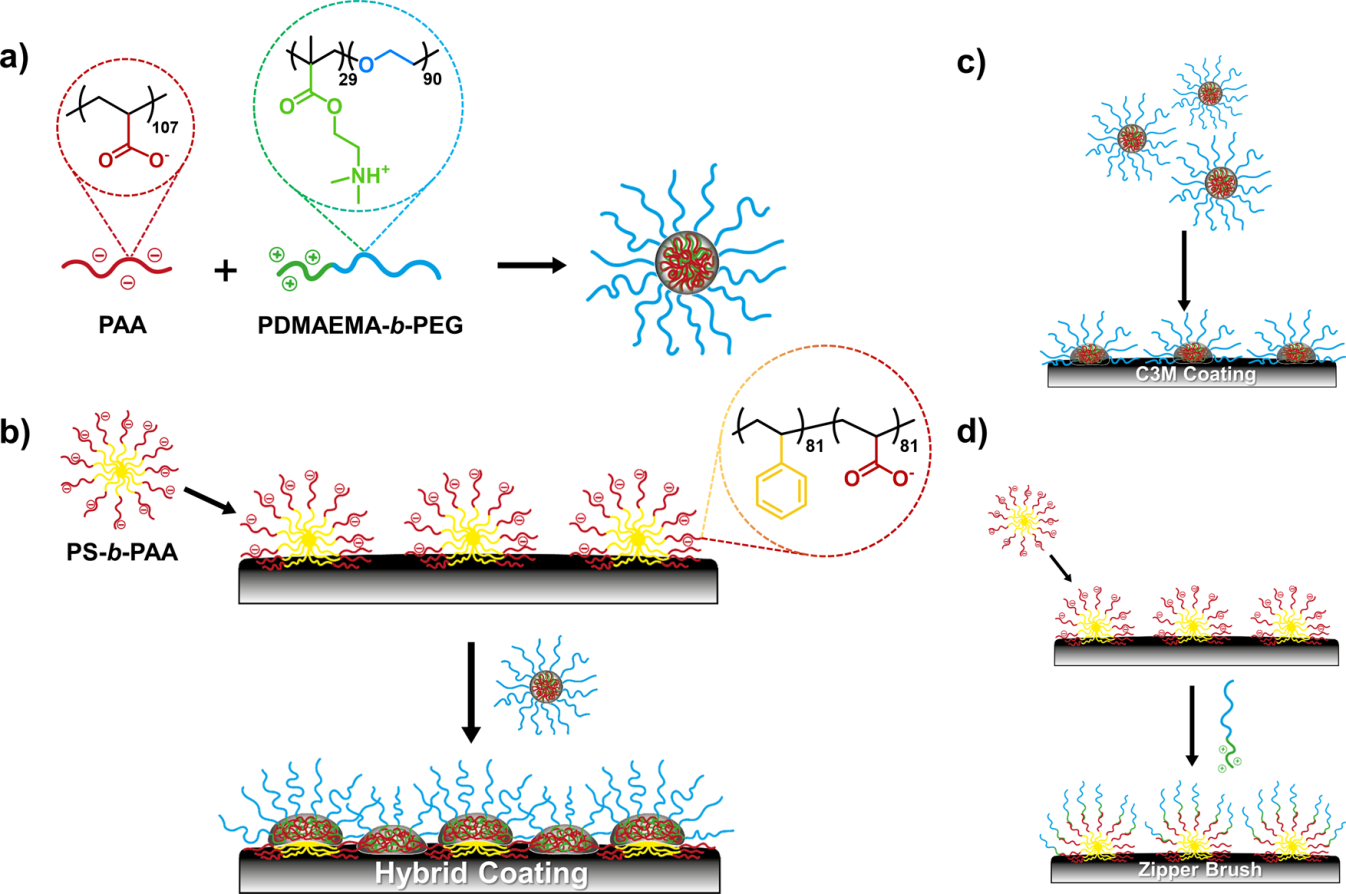
Schematic representation of the C3M formation and the polymer-based coatings obtained on hydrophobic surfaces via adsorption strategies. (a) The C3Ms self-assemble in aqueous solution by complexation of the negatively charged homopolymer (PAA) and the oppositely charged block (PDMAEMA) of the PDMAEMA-*b*-PEG diblock copolymer, resulting in spherical micelles. (b) The hybrid coating is obtained via a two-step adsorption procedure, which includes the initial adsorption of negatively charged PS-*b*-PAA micelles followed by complexation to pre-fabricated C3Ms. (c) The C3M coating is acquired through direct adsorption of C3Ms onto the PS substrate. (d) The formation of the zipper brush also requires an initial modification of the substrate by an adsorbed negatively charged micellar layer (PS-*b*-PAA), which is subsequently complexed to the PDMAEMA-*b*-PEG diblock copolymer.

Here, we combine the best of the C3M and zipper brush adsorption strategies to develop a new approach suitable for hydrophobic surfaces: a simple and reversible two-step adsorption process (Figure 1). This process is based on the initial adsorption of negatively charged polystyrene-*block*-poly(acrylic acid) (PS-*b*-PAA) micelles via their hydrophobic PS core followed by the subsequent complexation to pre-fabricated C3Ms (Figure 1a,b). We investigate the formation, layer characteristics, antifouling performance, and reversibility of the coating obtained via this two-step adsorption strategy while comparing it to the one-step adsorbed C3M coating (Figure 1c) and two-step adsorbed zipper brush (Figure 1d).

## 2 Experimental Section

### 2.1 Materials

2-(Dodecylthiocarbonothioylthio)-2-methylpropionic acid (DDMAT, 98%), 1,4-dioxane (≥ 99.0%), 2-(dimethylamino)ethyl methacrylate (DMAEMA, hydroquinone stabilized, 98%), 1,1,4,7,10,10-hexamethyltriethylenetetramine (HMTETA, 97.0%), azobisisobutyronitrile (AIBN, 98%), aluminum oxide (Al_2_O_3_, basic, activated), *N*,*N*-dimethylformamide (DMF, ≥99.9%), copper(I) bromide (CuBr, 98%), monopotassium phosphate (KH_2_PO_4_, ≥99.0%), bovine serum albumin (BSA, lyophilized powder, 66 kDa, ≥96%), lysozyme (lyophilized powder from chicken egg white, 14.3 kDa, ∼100,000 U mg^–1^), hydrogen peroxide (30% solution), deuterated chloroform (CDCl_3_, 99.8% D), and deuterated dimethylsulfoxide (DMSO-*d*_6_, 99.5% D) were purchased from Sigma-Aldrich. Hydrochloric acid (37–38% solution) and ammonia solution (25%, for analysis) were obtained from Boom B.V. Styrene (4-*tert*-butylcatechol stabilized, 99.0%) and sodium hydroxide pellets were purchased from Acros Organics. Methanol (≥99.9%), *n*-pentane (99%), isopropanol (≥99.8%), *n*-hexane (99%), and toluene (≥99.5%) were purchased from Macron Fine Chemicals. Absolute ethanol (99.9%) was purchased from J.T. Baker. Tetrahydrofuran (THF, BHT stabilized, ≥99.8%) and 1,1,1,3,3,3-hexafluoro-2-propanol (HFIP, ≥99.8%) were purchased from Biosolve. Poly(ethylene glycol) monomethylether (PEG_90_, *M*_n_ = 4.01 kg mol^–1^, *Đ* = 1.05) and *tert*-butyl acrylate (*t*BA, MEHQ stabilized, 98%) were purchased from TCI. Polystyrene (PS, *M*_n_ = 44.5 kg mol^–1^, *Đ* = 1.03, P19385-S) was purchased from Polymer Source, Canada. QCM-D sensors with a gold coating (Q-sense, QSX 301 Gold) were purchased from Quantum Design GmbH, Germany. Milli-Q was obtained from a Labconco WaterPro PS system in which water is purified four times: carbon filters then 2× deionization and then organic adsorption.

The 2-cyanopropan-2-yl propyl trithiocarbonate (CPP-TTC) chain transfer agent and PEG_90_-Br macroinitiator were synthesized as reported elsewhere.^50,51^

AIBN was recrystallized twice from methanol. Commercially available monomers were passed over a short aluminum oxide (basic) column to remove inhibitors prior to their direct use in the polymerizations. All other chemicals were used as received.

### 2.2 Sample Preparation

#### 2.2.1 Preparation of KH_2_PO_4_–NaOH Buffers

A 10 mM pH 8.0–8.1 phosphate buffer was prepared by dissolution of monopotassium phosphate (KH_2_PO_4_, 0.68 g) and 1.0 M NaOH (4.63 mL, i.e., 0.19 g of NaOH) in Milli-Q water (500 mL). All solutions in buffer were prepared using this specific phosphate buffer unless stated otherwise. To test the stability and reversibility of the adhered coatings, 500 mL stocks of buffers with the same pH but of higher ionic strengths were prepared: 50 mM (3.40 g KH_2_PO_4_, 0.94 g NaOH), 100 mM (6.80 g KH_2_PO_4_, 1.87 g NaOH), 500 mM (34.02 g KH_2_PO_4_, 9.36 g NaOH), and 1.0 M (68.05 g KH_2_PO_4_, 18.71 g NaOH). The buffer solutions were filtered (grade 15 filter paper) before use if required.

#### 2.2.2 Preparation of Polymer and Fouling Agent Solutions

All polymer and fouling agent stock solutions were prepared at least one day prior to the QCM-D measurement and were placed in a shaker to allow sufficient dissolution. For the zipper brush coating, the following polymer solutions were prepared: PS_81_-*b*-PAA_81_ (1.0 mg mL^–1^ in absolute ethanol) and PDMAEMA_29_-*b*-PEG_90_ (1.0 mg mL^–1^ in buffer). The fouling agent solutions included buffered solutions of bovine serum albumin (BSA, 1.0 mg mL^–1^) and lysozyme (1.0 mg mL^–1^). The solutions were filtered (grade 15 filter paper) before use if required.

#### 2.2.3 Preparation of Complex Coacervate Core Micelles (C3Ms)

The required C3Ms were obtained by one-step mixing of equal volumes of buffered solutions of the PAA_107_ homopolymer (0.5 mg mL^–1^) and PDMAEMA_29_-*b*-PEG_90_ diblock copolymer (3.0 mg mL^–1^) to obtain an *f_+_* mixing fraction of 0.5. The mixing fraction is defined by
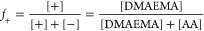
1where [+] and [−] denote the molar concentrations of positively and negatively chargeable monomers forming the micellar core, respectively. A fraction of 0.5 should facilitate a full charge compensation of negative and positive charges within the complexed core, rendering it charge-neutral.^45,52,53^ The polymer stock solutions were mixed dropwise using a syringe pump (ProSense, NE300) with a dispense rate of 300 μL min^–1^. To investigate the stability of the C3Ms against pH, a stock solution of C3Ms was prepared in 10 mM NaCl according to the procedure described above. The pH was adjusted by addition of aqueous NaOH and HCl stock solutions, and a DLS sample was measured at roughly every 0.5 pH unit interval. To investigate the stability of the C3Ms against salt, another stock solution of C3Ms was prepared in 10 mM NaCl without any adjustment of the pH, and after which, the salt concentration was slowly altered by mixing in stock solutions of higher ionic strength (10, 100, 500 mM, and 1.0 M NaCl). For each alteration in ionic strength, a DLS measurement was performed.

#### 2.2.4 QCM-D Sensor Cleaning

The QCM-D sensors (QSX 301 Gold, QuantumDesign, Germany) were thoroughly cleaned according to the protocol provided by Qsense: UV/ozone treatment (10 min); base piranha etching for 15 min at 75 °C in a 5:1:1 mixture of Milli-Q water (10 mL), ammonia (25%, 2 mL), and hydrogen peroxide (30%, 2 mL); cooling down in piranha solution (10 min); thorough rinsing with Milli-Q water; drying with nitrogen gas; UV/ozone treatment (10 min); and then immediate spin-coating with polystyrene. In case sensors were reused, an extra step was included at the start of the cleaning protocol, namely, soaking and sonication of the sensors in toluene (15 min) in order to remove the PS film and adsorbed polymer.

#### 2.2.5 Polystyrene (PS) Spin-Coating and Thermal Annealing

PS films were prepared by spin-coating a 0.45 μm filtered 1.5 wt % PS (*M*_n_ = 44.5 kg mol^–1^, *Đ* = 1.03) in toluene solution onto gold-plated QCM-D sensors (4000 rpm, 60 s). The thin films were thermally annealed in an oven for 20 min at 120 °C to reinforce its adhesion to the surface, and after which, the dry thickness was determined with ellipsometry (40.9 ± 0.2 nm).

### 2.3 Characterization

#### 2.3.1 pH Characterization

The pH of all aqueous solutions was measured using a pH meter (Mettler Toledo FiveEasy FP20, LE438 electrode). If required, the pH of the solutions was corrected by addition of NaOH (0.1 M and/or 1.0 M) and HCl (0.1 M and/or 1.0 M) solutions to obtain a consistent pH of 8.0–8.1.

#### 2.3.2 Quartz Crystal Microbalance with Dissipation (QCM-D)

To measure the mass and viscoelasticity of the adsorbed polymer layers, QCM-D measurements were performed on a four-channel Q-Sense E4 system connected to an Ismatec IPC peristaltic pump. AT-cut gold-plated quartz crystal sensors (QSX 301 Gold, QuantumDesign, Darmstadt, Germany) with a fundamental resonance frequency of 4.95 MHz and a diameter of 14 mm were thoroughly cleaned as described in Section 2.2 and subsequently spin-coated with a thin film of polystyrene. The sensors were then mounted in the QCM-D flow modules (QFM401), which were inserted in the analyzer and connected to the peristaltic pump. After equilibration in air (1 h) and the reference solution (1 h), the QCM-D response was recorded at the fundamental frequency (1st) and six different overtones (3rd, 5th, 7th, 9th, 11th, and 13th) at a temperature of 22 °C and constant flow rate of 150 μL min^–1^. The frequency response (*f*) of QCM-D includes the mass contributions from both the polymer and the water molecules within or bound to the polymer chains. A negative frequency shift (Δ*f*) indicates an increase in the adsorbed mass on the sensor surface. Changes in energy loss or dissipation (Δ*D*) represent the viscoelasticity of the polymer coating, which is related to the extent of hydration and conformation of the adsorbed polymers. In the case of dense or rigid layers, Δ*D* = 0, but for soft, non-rigid, or rough layers, the damping of the layer is significant and Δ*D* > 0.

Each step within QCM-D included cycled switching of the following solutions once stabilization was reached: reference solution (ethanol or buffer, to obtain a baseline); polymer, C3M, or protein solution (adsorption); and then reference solution (rinsing). Typically, the adsorbing solutions were flushed through the cell for at least 40 min. The polymer (1.0 mg mL^–1^), C3M (1.75 mg mL^–1^), and protein (1.0 mg mL^–1^) solutions were prepared at least one day in advance according to the methods described in Section 2.2. In order to test the stability and reversibility of the adhered coatings, the surfaces were rinsed with buffers of varying ionic strengths (50 mM, 100 mM, 500 mM, and 1.0 M) followed by the reference buffer solution (10 mM) once stabilization was reached. After each QCM-D measurement, the sensors were removed from the flow modules and dried carefully under a nitrogen flow. The dry layer characteristics were subsequently analyzed with AFM, VASE, and CA.

Due to the multilayer design of the polymer coatings, it was impossible to reliably model the data via available analysis software such as Q-tools, Dfind, or QTM. Instead, it was decided to focus on the qualitative data (Δ*f* and Δ*D*) rather than the quantitative data. To confirm reproducibility of the data, all adsorption experiments were performed at least six times.

#### 2.3.3 Atomic Force Microscopy (AFM)

The surface topology and roughness of the dried coatings were investigated using a Bruker Icon AFM system, equipped with a VTESPA-300 cantilever purchased from Bruker (42 N m^–1^, 300 kHz, tip radius = 5 nm). The AFM was operated using the standard tapping mode in air. AFM images were recorded with scan sizes ranging from 0.5 × 0.5 to 5 × 5 μm with scanning rates of 0.5 or 1 Hz and 256 samples/line (i.e., a pixel resolution of 256 × 256). For each scan size, images were recorded on at least three different spots on the coated sensor. The obtained raw images were processed using Gwyddion 2.55 software, employing specific processing steps regarding data leveling and background subtraction, including (1) level data by mean plane subtraction, (2) level data to make facets point upward, (3) aligning rows using a median of differences, (4) removing the polynomial background using a horizontal and vertical polynomial degree of one, (5) correcting for horizontal scars (strokes) if necessary, and (6) shifting the minimum data value to zero. The processed images were subsequently analyzed within the same software to determine the average root mean square (RMS) surface roughness (*S*_q_) of each surface, and the standard deviation was calculated over at least six AFM height images with varying scan sizes while taking into account the accuracy and the precision of the machine (±1 Å).

#### 2.3.4 Variable-Angle Spectroscopic Ellipsometry (VASE)

For the determination of the dry thickness of all films and coatings, ellipsometry spectra were recorded in air on a calibrated JAW V-VASE ellipsometer (J.A. Woollam Co., Inc.) on at least two different spots on the coated sensor. The measurements were performed in the spectral range of λ = 300–1700 nm and at two different angles of incidence with respect to the substrate normal (70° and 75°). The thickness was evaluated from the experimentally measured ellipsometric angles ψ and Δ using a multilayer model in the supplied software (WVASE32). The sensor surface was modeled using the Au_nk1_mat layer (100 nm), while the spin-coated polystyrene as well as the adsorbed coatings were modeled by an individual Cauchy layer with parameters *A*_n_ = fitted (PS = 1.54; adhered films = coupled Cauchy), *B*_n_ = 0.01, *C*_n_ = 0, and *k* = 0. The models were fitted using a specified spectral range of λ = 600–1700 nm. The average thicknesses and associated standard deviations were calculated based on the outcomes of these consecutive measurements and corresponding fits while taking into account the accuracy and precision of the machine (±0.1%).

#### 2.3.5 Contact Angle (CA) Measurement

The wettability of the dried coatings was investigated using a Dataphysics OCA 15EC contact angle analyzer using the sessile drop method at room temperature. Using an automated microsyringe, a Milli-Q water droplet with a volume of 2 μL was placed onto the samples, and a snapshot was taken with a camera. The static contact angles were calculated using SCA20_U software. At least three contact angles at different spots on each sample were measured and averaged in order to obtain a representative value.

#### 2.3.6 Dynamic Light Scattering (DLS)

To determine the hydrodynamic diameter and size distribution of the PS_81_-*b*-PAA_81_ micelles and C3Ms, DLS measurements were performed on a Malvern Panalytical Zetasizer Ultra equipped with a helium-neon laser (λ = 633 nm) and an Avalanche Photodiode detector. PS_81_-*b*-PAA_81_ (1.0 mg mL^–1^ in absolute ethanol) and C3M (1.75 mg mL^–1^ in buffer) solutions were transferred to 10 × 10 mm quartz cuvettes without prior filtering. The samples were recorded fivefold in the back-scattering mode at 25 °C after a 120 s equilibration time. Results were analyzed using ZS Xplorer software. The average particle size and mean standard deviations were calculated based on these five consecutive measurements while taking into account the accuracy and the precision of the machine (±2%).

#### 2.3.7 Zeta Potential (ζ) Measurements

To study the complexation of the C3Ms, ζ-potential measurements were performed on a Malvern Panalytical Zetasizer Ultra system equipped with a helium-neon laser (λ = 633 nm) and an Avalanche photodiode detector. The C3M solution was transferred to a disposable folded capillary zeta cell, and the measurements were performed at 25 °C. Samples were recorded thrice with a maximum of 30 cumulative recordings. Results were analyzed using ZS Xplorer software. The average zeta potential and corresponding mean standard deviation was calculated based on these three consecutive measurements while taking into account the accuracy and the precision of the machine (±2%).

#### 2.3.8 Transmission Electron Microscopy (TEM)

TEM imaging of the self-assembled and complexed micelles was performed on a Philips CM120 transmission electron microscope using a LaB_6_ filament and operated at an accelerating voltage of 120 kV. Images were recorded using a Gatan 4 k CCD camera. TEM grids (copper, 400 mesh with a carbon support film) were glow-discharged prior to sample preparation (15 s at 40 mA and 300 V). Specimens were prepared by deposition of 5 μL of the micellar solution (*c* = 0.3 g L^–1^ in ethanol for PS-*b*-PAA micelles and *c* = 0.6 g L^–1^ in buffer for C3Ms) onto the grid and adsorption for 1 min before blotting. Before the specimen was fully dried, 5 μL of 2 wt % uranyl acetate staining solution was deposited onto the grid; this was immediately blotted, and a new 5 μL drop of staining solution was deposited and left to adsorb for 1 min before blotting. TEM images were analyzed using ImageJ software, employing brightness and contrast correction tools to enhance the general quality of the snapshots and calculate the average particle size (including its mean standard deviation).

#### 2.3.9 Proton Nuclear Magnetic Resonance (^1^H NMR) Spectroscopy

^1^H NMR spectra were recorded on an Agilent 400-MR 400 MHz spectrometer operating at room temperature. Polymer samples were dissolved in the appropriate deuterated solvent with a concentration of approximately 20 mg mL^–1^. The resulting ^1^H NMR spectra were analyzed using MestreNova software (version 14.2.0).

#### 2.3.10 Gel Permeation Chromatography (GPC)

To determine the relative molecular weights and the molar mass distributions of the synthesized polymers, GPC was performed in DMF (containing 0.01 M LiBr) on a Viscotek GPCMax system equipped with model 302 TDA detectors and two columns (PolarGel L and M, 8 μm 30 cm) from Agilent Technologies at a flow rate of 1 mL min^–1^. The columns and detectors were maintained at a temperature of 50 °C. Near monodisperse poly(methyl methacrylate) standards from Polymer Standards Service were used for the construction of a calibration curve based on conventional calibration. All polymer samples were dissolved in DMF-LiBr (concentration ≈ 2–3 mg mL^–1^) at least one day in advance and were passed through a 0.20 μm PTFE filter prior to injection. Data acquisition and calculations were performed using Viscotek Omnisec software (version 5.0).

#### 2.3.11 Attenuated Total Reflection–Fourier Transform Infrared (ATR-FTIR) Spectroscopy

ATR-FTIR spectra were recorded on a Bruker VERTEX 70 spectrometer equipped with an ATR diamond single reflection module. The spectra were collected in the range of 4000–400 cm^–1^ with a spectral resolution of 2 cm^–1^ and using 64 scans for each sample. Atmospheric compensation and baseline corrections (concave rubberband correction, 10 iterations) were applied to the collected spectra using Bruker’s OPUS spectroscopy software (version 7.5).

## 3 Results and Discussion

### 3.1 Polymer Synthesis

The poly(acrylic acid) homopolymer (PAA_107_, *M*_n_ = 13.4 kg mol^–1^, *Đ* = 1.14) and polystyrene-*block*-poly(acrylic acid) diblock copolymer (PS_81_-*b*-PAA_81_, *M*_n_ = 14.6 kg mol^–1^, *Đ* = 1.15) were successfully synthesized through reversible addition–fragmentation chain transfer (RAFT) polymerization. The purchased poly(ethylene glycol) homopolymer (PEG_90_, *M*_n_ = 4.01 kg mol^–1^, *Đ* = 1.05) was converted into the PEG_90_-Br macroinitiator, and after which, it was chain-extended with 2-(dimethylamino)ethyl methacrylate) (DMAEMA) through atom transfer radical polymerization (ATRP) to obtain poly(2-(dimethylamino)ethyl methacrylate)-*block*-poly(ethylene glycol) (PDMAEMA_29_-*b*-PEG_90_, *M*_n_ = 13.8 kg mol^–1^, *Đ* = 1.22). All polymers were obtained in high purity and with low dispersities as was evidenced by ^1^H NMR, GPC, and ATR-FTIR. The detailed experimental procedures and characterization of the synthesized polymers are included in the Supporting Information.

### 3.2 Polymer Behavior in Solution

#### 3.2.1 Self-Assembly of PS-*b*-PAA

**Figure 2:**
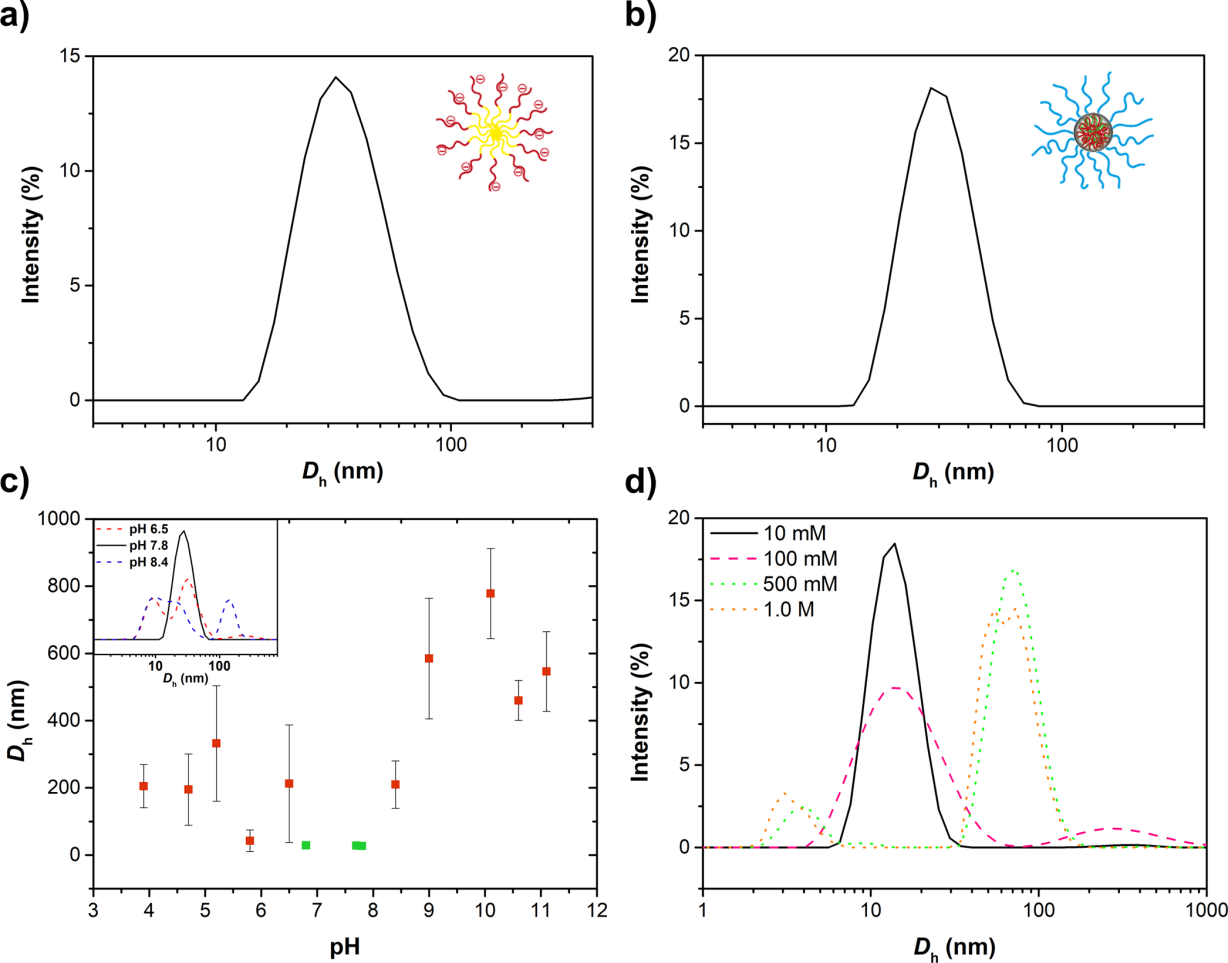
Size distributions of (a) the self-assembled PS-*b*-PAA micelles and (b) C3Ms (PAA + PDMAEMA-*b*-PEG), including the stability of C3Ms versus the (c) pH and (d) salt concentration. The C3Ms were considered unstable once the DLS data identified multiple populations, which occurred outside the optimum regime of pH = 6.5–8.4 and above a salt concentration of 100 mM. The error bars in (c) represent the standard deviations of the mean *D*_h_ values.

Due to dispersion issues of PS-*b*-PAA in buffer, it was decided to disperse the diblock copolymer in ethanol instead, which is a selective solvent for PAA. This facilitated its self-assembly into micelles consisting of a hydrophobic PS core and a hydrophilic PAA corona as was evidenced by dynamic light scattering (DLS) (Figure 2a). The micelles are monodisperse with an average hydrodynamic diameter of *D*_h_ = 35.1 ± 0.7 nm and a polydispersity index (PDI) of 0.24. The PS-*b*-PAA micelles were additionally characterized with TEM, which further confirmed the formation of small and monodisperse micelles (Figure S15a).

#### 3.2.2 Self-Assembly and Stability of C3Ms

The required C3Ms were obtained by mixing buffered solutions of the negatively charged PAA homopolymer and cationic-neutral PDMAEMA-*b*-PEG diblock copolymer at a charge stoichiometric ratio (Figure 1a). This should facilitate full charge compensation of the negative and positive charges within the complexed core thereby rendering it charge-neutral.^27,52,53^ The highly monodisperse micelles are characterized by an average hydrodynamic diameter of *D*_h_ = 28.2 ± 0.6 nm and a PDI of 0.09 (Figure 2b). The formation of monodisperse C3Ms was additionally verified by TEM (Figure S15b). The measured zeta potential (ζ = 0.02 ± 1.12 mV) is nearly zero, which confirms the formation of charge-neutral C3Ms.

The stability of the formed C3Ms was tested against both the pH and salt concentration. The system was considered unstable once the DLS data identified multiple populations. PAA (p*K*_a_ = 4.5) and PDMAEMA (p*K*_a_ = 7.8) are both weak polyelectrolytes, so their net charge varies with the pH.^54,55^ It is therefore to be expected that the micelles only self-assemble in a narrow regime, namely, from pH = 6.5 to pH = 8.4 (Figure 2c). When adjusting the pH outside this range, the C3Ms disintegrate due to a decreased charge density of one of the polyelectrolyte blocks. However, when adjusting the pH back to the optimum regime, the well-defined micelles readily reform (Figure S16 and Table S1). Considering the stability against salt, it was found that the C3Ms lose their well-defined monodisperse structure at an ionic strength of 100 mM NaCl or higher (Figure 2d), which once again demonstrates the sensitivity of the system at hand. Such a high sensitivity to salt has been reported before for C3M systems.^48^ There are many ways to increase the stability of C3Ms, namely, by careful tuning of the corona/core length ratio, increasing the hydrophobicity of the polyelectrolyte blocks, increasing either or both polyelectrolyte block lengths, or by cross-linking the coacervate core.^44,48,56−58^ However, we decided to take advantage of this sensitivity feature in order to endow the hybrid coating with a salt-triggered reversibility property.

### 3.3 Coating Formation

The adsorption of the individual diblock copolymers and C3Ms onto a surface was monitored in situ by means of a quartz crystal microbalance with dissipation (QCM-D). The gold QCM-D sensors were rendered hydrophobic by spin-coating a thin polystyrene film (40 nm) on top. Within the QCM-D, both the frequency response (Δ*f*) and changes in energy dissipation (Δ*D*) were recorded. The first relates to the mass adsorbed to the QCM-D sensors, while the latter represents the rigidity of the formed polymer coating. Simply stated, when the dissipation is (close to) zero, the film is relatively rigid, but when the dissipation increases, the film has a more viscous and hydrated character.

Three types of adsorbed coatings were produced within the QCM-D: the one-step C3M coating, the two-step zipper brush, and the two-step hybrid coating (Figure 1). The one-step process relies on the direct adsorption of C3Ms formed between the PAA homopolymer and PDMAEMA-*b*-PEG diblock copolymer (Figure 1a). They adsorb via their pseudo-hydrophobic charge-neutral core, while the hydrophilic and antifouling PEG corona extends into the solution (C3M coating; Figure 1c).^43,45^ The two-step adsorption process is based on the initial adsorption of negatively charged PS-*b*-PAA micelles via their hydrophobic PS core followed by the subsequent complexation to either PDMAEMA-*b*-PEG (zipper brush; Figure 1d) or pre-fabricated C3Ms (hybrid coating; Figure 1b). The block lengths and block ratios of the selected polymers were carefully tuned in order to optimize the stability and the surface density of the obtained coatings. The size of the PS block (PS-*b*-PAA) allows for a sufficiently strong interaction with the hydrophobic surface while preventing extensive crowding. The density of the resulting micellar layer is subsequently maximized by complexation to either the C3Ms or PDMAEMA-*b*-PEG diblock copolymer. In the latter case, the density is additionally enhanced by incorporating a multiplication factor of approximately 3, so multiple PDMAEMA blocks can bind to a single PAA chain.^24^ In the case of the C3Ms, the 1:3 block ratio of the charged and neutral block comprising the diblock copolymer enhances the stability of the C3Ms and prevents them from growing to macroscopic dimensions.^46^ Moreover, the inclusion of a weak polyelectrolyte complex endows the hybrid coating with an integrated reversibility property: by addition of salt, the charge-complexed cores disassemble, which allows for an easy removal of the (fouled) top layer without the need for aggressive cleaning agents. Subsequent recoating via a one-step procedure permits the preparation of a new and fully functional antifouling coating. Hence, long-term chemical stability and antifouling durability are no longer a requisite as the straightforward applicability and reversibility allow facile regeneration of the coating.

**Figure 3:**
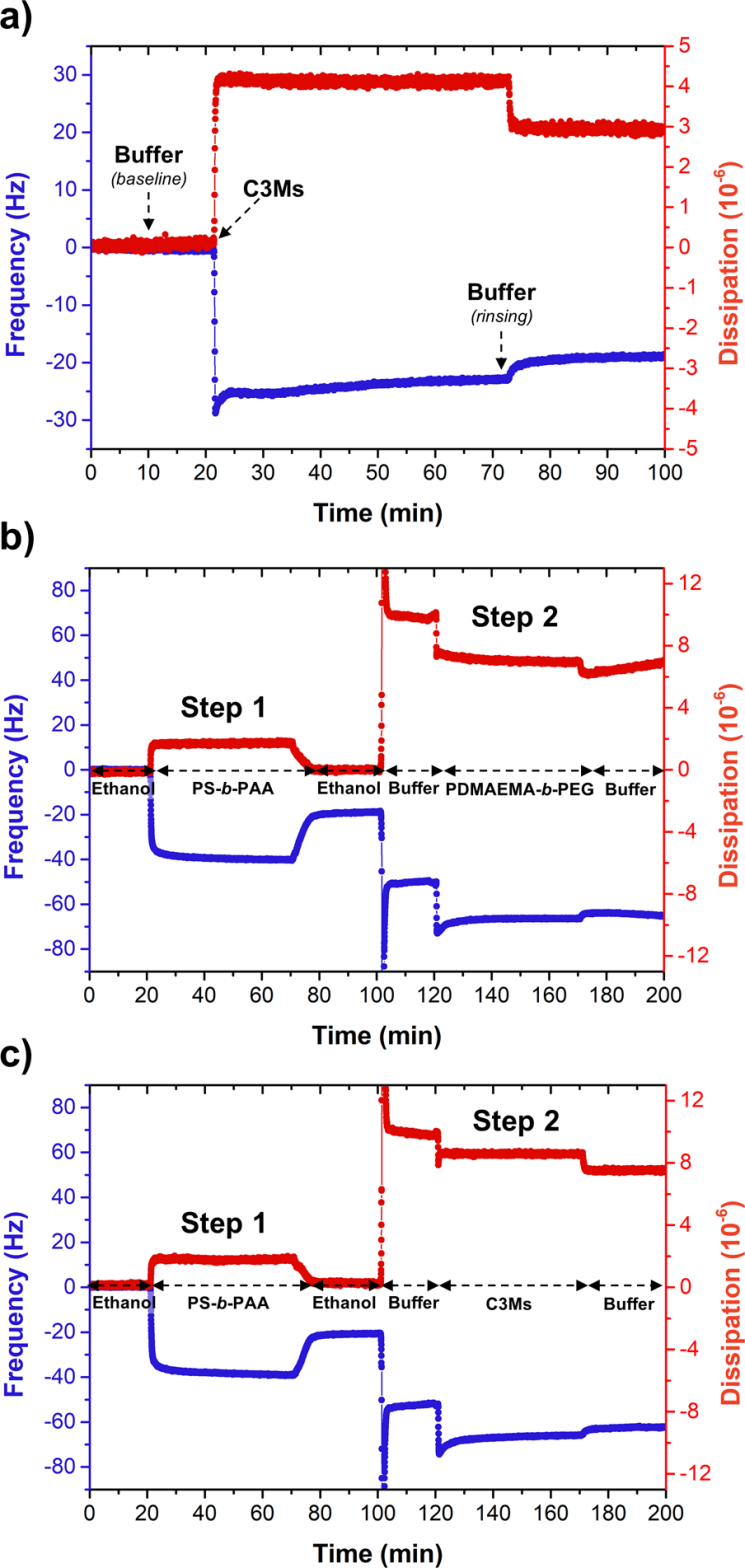
QCM-D graphs showing the in situ formation of the (a) C3M coating, (b) zipper brush, and (c) hybrid coating. Each adsorption step can be characterized by three stages: an initial rapid adsorption regime (negative frequency shift) followed by an adsorption equilibrium (plateau) and finally a mass loss of loosely attached or unbound material during rinsing (positive frequency shift). Even though the PS-*b*-PAA primer layer is relatively rigid (net zero dissipation), all final coatings can be identified as thin viscous films as is indicated by the net decrease in the frequency and positive dissipation shift. For the sake of clarity, all harmonic overtones were omitted except for R5, depicting the total shifts in both the frequency (F5, blue) and energy dissipation (D5, red). A QCM-D graph including all harmonic overtones can be found in Figure S17.

The adsorption data of the one-step adsorbed C3M coating is shown in Figure 3a. The adsorption kinetics of the C3Ms onto the PS-coated sensor can be divided into three distinct regimes: an initial rapid adsorption of C3Ms to the sensor upon introduction into the cell followed by an adsorption equilibrium (plateau) due to an increased energy barrier and finally a mass loss of loosely attached or unbound C3Ms when rinsed with the reference buffer solution. A minor frequency change of approximately 17% and the net positive dissipation after rinsing suggest the formation of a strongly bound but viscous C3M coating (Δ*f*_C3M_ = −17 Hz, Δ*D*_C3M_ = 2.5). A similar adsorption behavior was reported by Hofs et al. who also confirmed secured binding of their PEG-based C3Ms to various surfaces.^27^

Both the zipper brush and hybrid coating involve a two-step adsorption process, starting with the adhesion of PS-*b*-PAA micelles from ethanol (Figure 3b,c, step 1). The adsorption kinetics are similar to the one seen for the C3M coating, but more loosely bound material is removed during rinsing (approximately 48%) and the dissipation shifts back to zero, suggesting the formation of a rigid film (Δ*f*_primer_ = −20 Hz, Δ*D*_primer_ = 0). The reference solution was subsequently switched to buffer and a solution of either PDMAEMA-*b*-PEG (zipper brush) or pre-fabricated C3Ms (hybrid coating) entered the system (Figure 3b,c, step 2). The adsorption kinetics are almost identical to each other and can again be characterized by a fast adsorption regime, an equilibrium region, and the removal of weakly attached material (Δ*f*_zip_ = −17 Hz, Δ*D*_zip_ = −3.4, and Δ*f*_hybrid_ = −12 Hz, Δ*D*_hybrid_ = −2.2). Interestingly, the complexation step is defined by a negative dissipation shift, which could be explained by a loss of flexibility and the release of bound counterions and water molecules when the PDMAEMA-*b*-PEG polymers or C3Ms penetrate and complex to the stretched out PAA chains.^59^ In the case of the C3Ms, it is envisioned that some PAA homopolymers may leach out of the core during complexation, so the coating remains charge-neutral. This may provide the hybrid coating with an increased stability and enhanced surface density in contrast to the hydrophobically attached C3M coating. The small frequency change after rinsing (approximately 21%) suggests a strong interaction between the two distinct layers.

### 3.4 Coating Characterization

**Figure 4:**
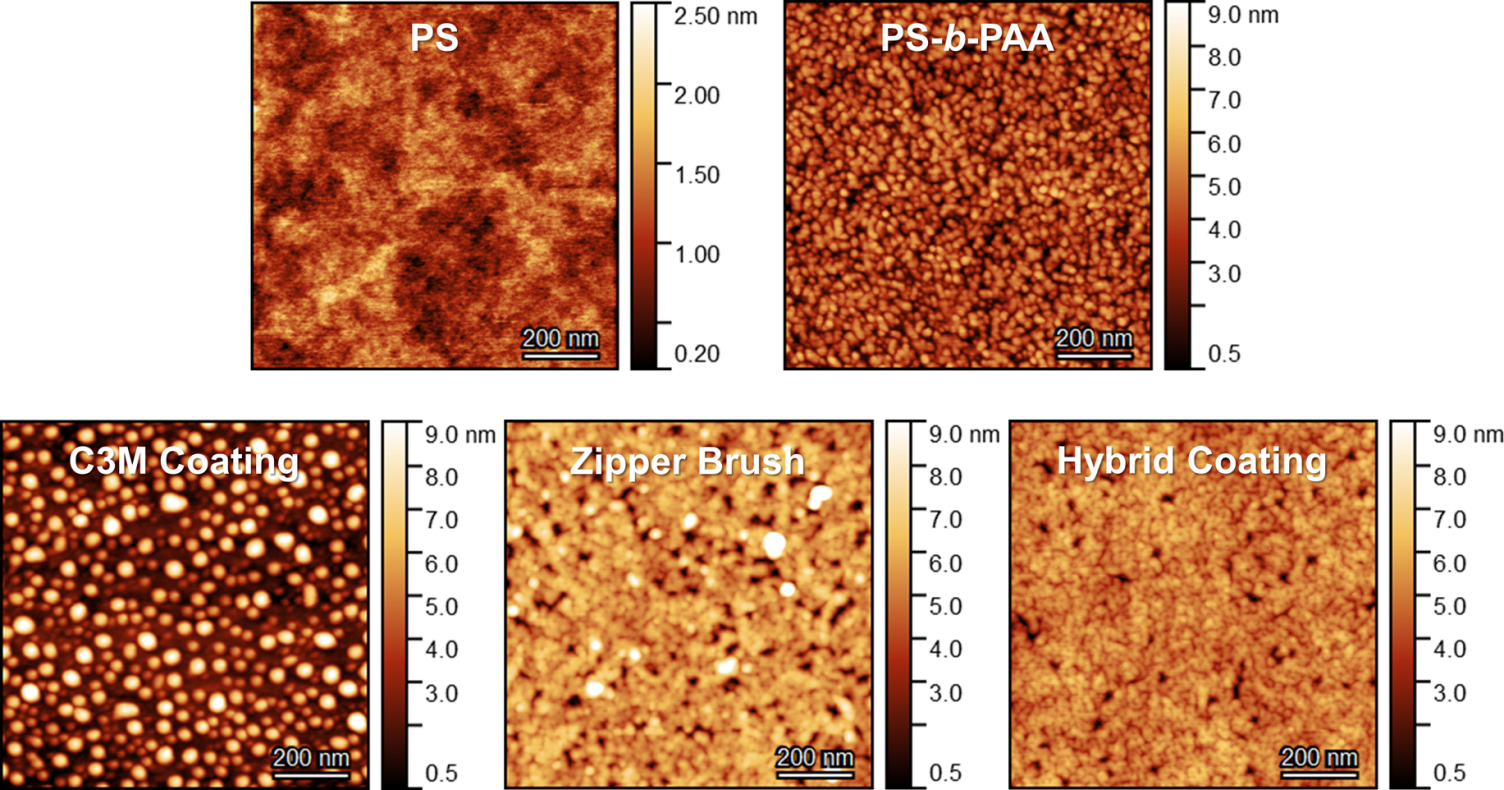
AFM height images of the three adsorbed polymer-based coatings, including the hydrophobic polystyrene surface and PS-*b*-PAA primer. The AFM images were recorded using standard tapping mode in air. The corresponding phase images and cross-sectional profiles are available in Figures S18 and S19.

After the in situ formation of the adsorbed polymer-based coatings within QCM-D, each of them was subsequently characterized and compared based on surface topography, thickness, and wettability (Figure 4 and Table 1) using AFM, ellipsometry, and contact angle measurements, respectively.

**Table 1:** Summarized Characterization Data of the Coated Surfaces, Including the Dry Thickness Measured with Ellipsometry, the Root Mean Square (RMS) Surface Roughness (*S*_q_) Obtained from Tapping-Mode AFM, and the Recorded Static Contact Angles (θ) in Water^a^

coating	thickness (nm)	*S*_q_ (nm)	θ (°)
PS	40.9 (± 0.2)	0.3 (± 0.1)	93.2 (± 0.7)
PS-*b*-PAA	1.8 (± 0.1)	1.3 (± 0.1)	65.4 (± 3.5)
C3M coating	1.4 (± 0.1)	1.9 (± 0.1)	77.8 (± 0.2)
zipper brush	4.2 (± 0.1)	1.5 (± 0.1)	56.9 (± 2.9)
hybrid coating	4.0 (± 0.1)	0.8 (± 0.1)	38.3 (± 1.1)

a The captured contact angle images can be found in Figure S20. Each denoted value represents the mean ± standard deviation calculated based on at least three different spots on the coated sensors while taking into account the accuracy and precision of each analysis method.

The QCM-D sensors were successfully coated with a uniform thin film of hydrophobic polystyrene (θ = 93°) characterized by a dry thickness of approximately 40 nm and a low surface roughness of just 0.3 nm. Subsequent adsorption of self-assembled C3Ms yielded a 1.4 nm-thick C3M coating with a pronounced micellar topography (Figure 4). The micelles spread and cover the underlying polystyrene film but do so without unfolding into a brush, resulting in a low-density film with a relatively high surface roughness (1.9 nm). The flattened spheres have a height of approximately 8 ± 2 nm, which is much smaller than their hydrodynamic diameter in solution (approximately 28 nm). This would suggest that the C3Ms adsorb via their coacervate core rather than via their PEG corona, as was expected. However, since the AFM images were recorded in air, the flattened structure may also (partly) be the result of drying as the solvent contained in the C3Ms leaches out and evaporates. Due to an insufficient surface coverage by the flattened micelles, the underlying PS film affects and even dominates the overall wettability as is indicated by the remarkably higher contact angle (78°) compared to what was initially expected for a PEG-coated surface (36–39°).^60^ The topography and wettability of this adsorbed C3M coating is highly similar to the PEG-based C3M coating reported by Hofs et al.^27^ Their C3Ms also adsorbed as intact and flattened single micelles, resulting in a low-density coating with a relatively high advancing contact angle of 69°.^27^

The PS-*b*-PAA micelles adsorbed into a 1.8 nm-thick film without unfolding into a brush as is evident from the micellar topography seen in AFM (Figure 4). The modified surface exhibits a more hydrophilic character than before (from 93° to 65°) with a wettability resembling that of a PAA-based film (57–73°).^61−63^ This confirms the anticipated conformation of the adsorbed micelles: the PS cores adsorb to the surface, forcing the PAA chains to stretch outward. This is a promising result as only a correct conformation of the PAA chains allows complexation to the second (antifouling) layer.

Complexation of PDMAEMA-*b*-PEG to the PS-*b*-PAA primer increased the coating thickness from 1.8 to 4.2 nm as well as the surface roughness (from 1.3 to 1.5 nm). The micellar topography transitioned into a denser surface structure, indicating that the zipper brush formation was successful (Figure 4). However, the film does not cover the surface uniformly as is reflected by the random gaps and taller features. In addition, the measured contact angle (57°) is higher than is expected for a PEG-based film (36–39°).^60^ Instead, the wettability rather represents an intermediate between a PEG film and a PDMAEMA film (65°).^64^ This could suggest an incorrect conformation of several PDMAEMA-*b*-PEG polymer chains during complexation in which PEG chains interact with the pre-adsorbed PAA chains via hydrogen bonding thereby positioning the positively charged PDMAEMA chains at the top (Figure S21).^65^

The topography of the 4.0 nm-thick hybrid coating resembles the one of the zipper brush albeit with a smaller surface roughness of 0.8 nm. This is not surprising, considering both coatings consist of similar components and showed identical adsorption behavior within the QCM-D. However, this coating has a noticeably higher wettability with a contact angle (43°) comparable to that of a PEG-based film. Since the C3Ms reproducibly self-assemble into identical structures with a coacervate core and a PEG corona, the final morphology of the hybrid coating is more easily controlled: the PEG chains will always protrude outward into the solution, away from the coated surface.

### 3.5 Antifouling Performance

The antifouling performance of the adsorbed coatings was tested against two types of fouling agents with varying characteristics, namely, bovine serum albumin (BSA) and lysozyme. Under current conditions (pH = 8.0), BSA can be described as a flexible and hydrophilic protein with an overall negative charge, while lysozyme is a twice as small hydrophilic enzyme with a net positive charge.^66,67^ As a control experiment, adhesion of the two fouling agents was also tested on the pristine PS-coated substrate (Figure S22) and the PS-*b*-PAA primer (Figure S23). While both lysozyme and BSA adsorbed at similar extents to PS, only lysozyme was able to adhere strongly to the negatively charged PS-*b*-PAA primer as was expected based on electrostatics.

**Figure 5:**
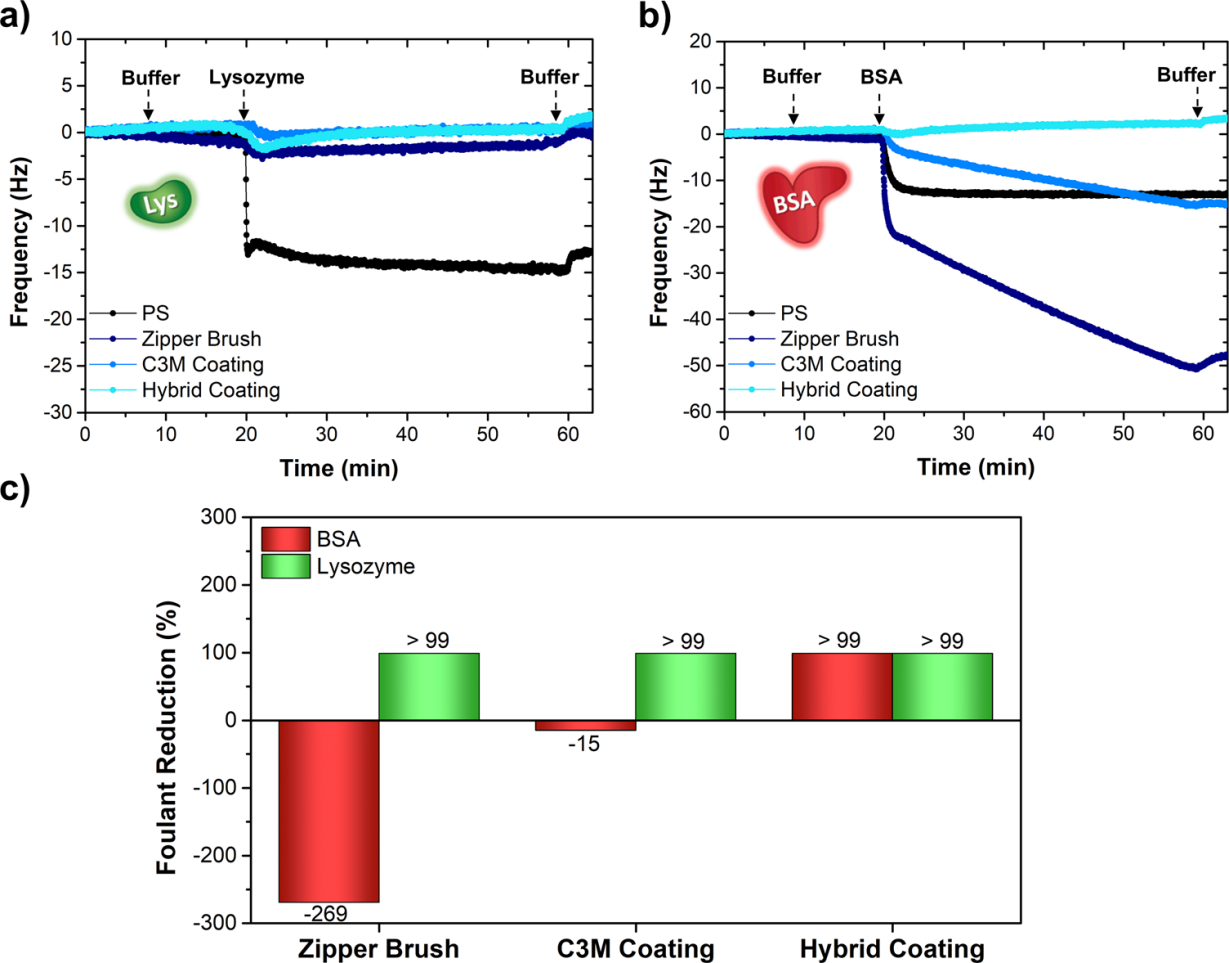
QCM-D graphs summarizing the in situ antifouling performance of the coated surfaces against (a) lysozyme and (b) BSA. Within the margin of error of the QCM-D, it can be concluded that all coatings prevent the attachment of lysozyme, but only the hybrid coating can effectively suppress BSA adhesion. For the sake of clarity, all harmonic overtones were omitted except for F5. (c) Bar graph representing the antifouling efficiency of the three adsorbed polymer-based coatings with respect to the pristine PS-coated substrate.

To test the antifouling efficiency of the three polymer-based coatings, each one was reproduced within the QCM-D, and after which, lysozyme (Figure 5a) or BSA (Figure 5b) entered the system. In order to remove the weakly bound material, the surfaces were rinsed with the reference buffer solution once an equilibrium state was reached. According to the QCM-D data, all three adsorbed coatings successfully suppressed the attachment of lysozyme (Δ*f* = 0) as opposed to the pristine polystyrene substrate (Figure 5a and Figure S24). However, only the hybrid coating was able to effectively suppress the adhesion of both fouling agents as is represented by its unaffected frequency signals (Figure 5b). When normalizing the frequency shifts with regard to the pristine PS-coated substrate and converting the deviations into a bar graph, the differences in antifouling performance become even more striking (Figure 5c). While the C3M coating and zipper brush dramatically suppress the attachment of lysozyme, BSA is able to adhere more strongly to these films than to the pristine PS-coated substrate. Contrastingly, the hybrid coating effectively prevents the attachment of both lysozyme and BSA.

**Figure 6:**
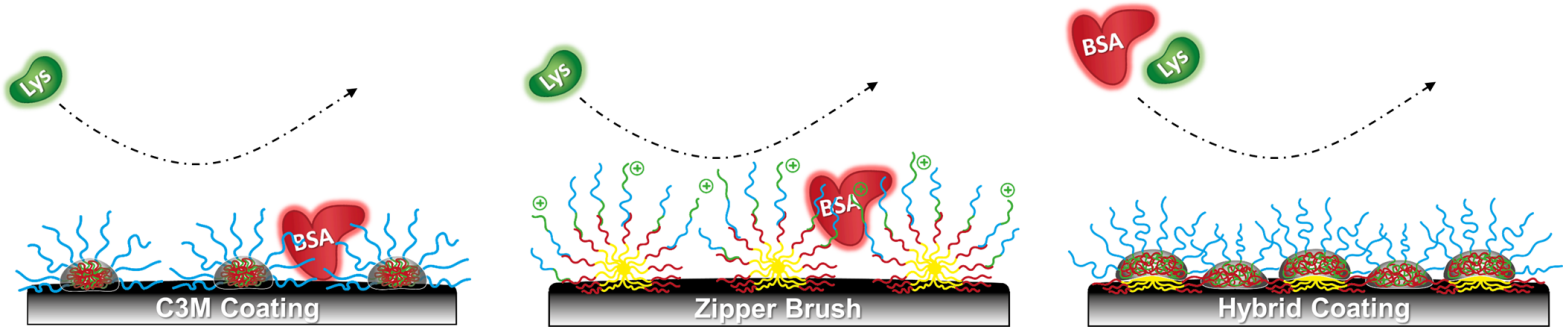
Schematic representation of the hypothesized topology and antifouling character of each adsorbed polymer-based coating. The low surface coverage of the C3M coating and the incorrect chain conformation of the zipper brush permit the adhesion of BSA, while the high-density and hydrophilic hybrid coating effectively inhibits it.

Considering the striking differences in the antifouling efficiency, a slightly adapted schematic representation of the coatings’ topology and corresponding antifouling character is summarized in Figure 6. The imperfect antifouling performance of the C3M coating can be understood from its poor surface wettability and relatively low surface coverage, which provides many attachment sites for passing fouling agents. In the case of the zipper brush, a lack of control over the final conformation of the complexing polymer chains may cause the PEG chains to interact with the pre-adsorbed PAA chains via hydrogen bonding thereby positioning the positively charged PDMAEMA chains at the top.^65^ This gives rise to a net positive surface charge, which evidently strongly attracts negatively charged BSA but repels positively charged lysozyme. The zipper brush could be optimized by a change in the antifouling block, which may guide the complexation into the desired morphology. Considering the hybrid coating, such a conformational issue does not exist as the PEG chains consistently form the micelle corona, and the resulting high wettability provides the surface with an excellent antifouling property. In addition, the small size of the C3Ms may allow them to perfectly fit inside the cavities left by the micellar PS-*b*-PAA primer, resulting in a final coating with a higher surface density, which further facilitates its antifouling character. The unaffected surface topography after fouling testing additionally validates the remarkably efficient antifouling property of this film (Figure S25). Such an excellent fouling control has not yet been reported for neither covalently attached, nor adsorbed PEG-based coatings fabricated on hydrophobic surfaces (Table S2).^27,29,47,68−70^ Even though de Vos et al. reported similar antifouling performances for their zipper brush, the time-consuming Langmuir–Blodgett technique required to make the brush complicates its use for large-scale applications.^24,49^ The two-step hybrid approach does not include such a scale-limiting factor, which consequently makes it a superior strategy.

### 3.6 Stability and Reversibility of Hybrid Coating

Even though the hybrid coating showed a superior antifouling performance against both BSA and lysozyme, it may not maintain its antifouling durability in more complex environments. Due to the high variety of fouling agents, it is almost impossible to design an antifouling coating that permanently repels all of them on a long-term basis. For this reason, the hybrid coating was designed to have a built-in easy-to-clean feature: a weak polyelectrolyte complex. A simple salt trigger should facilitate the dissociation and release of the C3M top layer and subsequent recoating would lead to a complete recovery of the antifouling properties.

To investigate this sacrificial regeneration strategy, the stability of the C3M top layer was tested against buffer solutions of varying ionic strengths. To verify that the PS-*b*-PAA primer would not interfere with the reversibility assessment, preliminary tests were carried out to investigate the stability of these films in solutions of higher ionic strengths. According to the QCM-D data, the adsorbed PS-*b*-PAA primer remained stable up to the highest salt concentration of 1.0 M (Figure S26 and Table S3).

**Figure 7:**
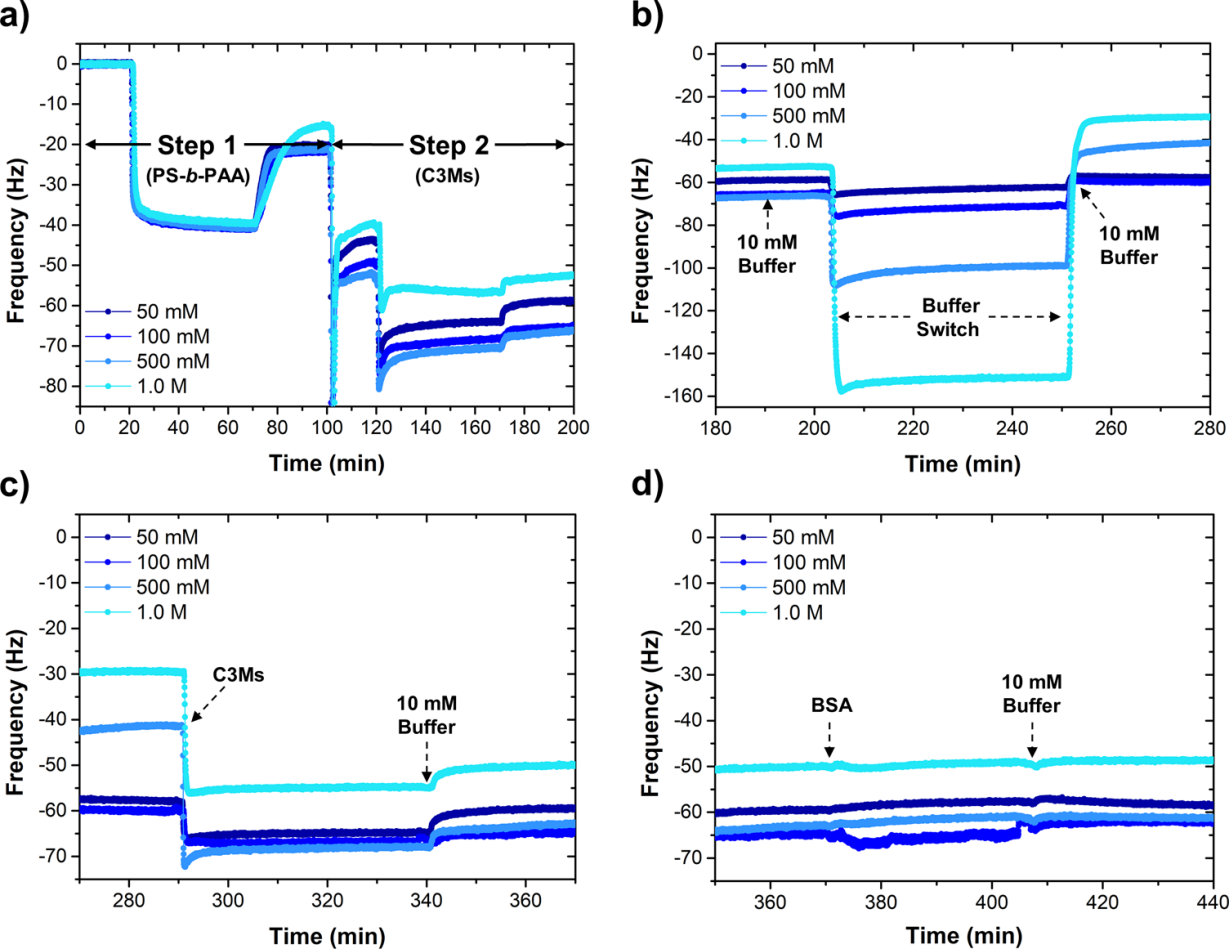
QCM-D graphs showing (a) the highly reproducible formation of the hybrid coating, (b) the response of the hybrid coating to buffers of varying ionic strengths, (c) the subsequent regeneration of the top C3M layer (in 10 mM buffer), and (d) the full recovery of the antifouling performance against BSA. Each salt-treated coating could be completely recovered, independent of the ionic strength used. For the sake of clarity, only the F5 harmonic overtone is shown and the dissipation signals are omitted.

Next, highly reproducible hybrid coatings were generated on top of PS-coated sensors (Figure 7a and Figure S27) within the QCM-D, which were subsequently exposed to buffer solutions of varying ionic strengths, ranging from 50 mM up to 1.0 M (Figure 7b). The frequency shifts that followed are predominantly related to a change in the buffer viscosity and density. After 50 min, the films were rinsed with the reference buffer (10 mM). From the net frequency changes, it can be concluded that only the 500 mM and 1.0 M buffers can completely disassemble and release the C3M layer from the surface. A 100 mM buffer solution seems to slightly weaken the complex (as was expected from DLS; Section 3.2.2), while the 50 mM buffer solution leaves it unaffected.

To repair or regenerate the antifouling layer, a 10 mM buffered solution of C3Ms was flushed over the salt-treated films, forcing the C3Ms to complex to the (partially) re-exposed PS-*b*-PAA primer (Figure 7c). Similar adsorption kinetics were recorded as observed before, and the frequency signals all moved back to their initial values seen prior to the salt treatment, indicating a successful renewal. Finally, to test the recovery of the antifouling performance, the recoated substrates were exposed to a solution of BSA (Figure 7d). The negligible change in the frequency confirms the excellent suppression of BSA, independent of the ionic strength of the buffer used. Hence, this hybrid film is a perfect example of a highly effective and reversible block copolymer-based antifouling coating.

## 4 Conclusions

By combining the previously reported one-step C3M and two-step zipper brush approaches into a new, simple, and reversible two-step adsorption strategy, we managed to develop a unique hybrid coating on hydrophobic surfaces with superior antifouling properties. All three adsorbed coatings managed to dramatically suppress the adhesion of lysozyme, but only the hybrid coating effectively prevented the attachment of BSA. We believe that this can be explained by its superior surface density and uniformity as well as its high wettability owing to the proper conformation of the PEG chains. In addition, the successful triggered release (>500 mM) and regeneration of the top C3M layer inside the hybrid coating led to a full recovery of its antifouling performance against BSA. Since the PS-*b*-PAA primer remains intact at these high ionic strengths, this two-step adsorbed sacrificial coating has the advantage of only needing to re-apply one layer instead of having to replenish the complete coating when fouled. The straightforward application strategy combined with its triggered renewability makes this hybrid coating a simple, cost-effective, eco-friendly, and therefore attractive alternative to existing (covalently grafted) antifouling coatings.

Further research is required to investigate and optimize the long-term chemical and mechanical stability as well as the antifouling durability of this promising hybrid coating and its realization in large-scale applications (e.g., via spray painting or dip-coating). Since the formation of the hybrid coating involves the complexation of two weak polyelectrolytes, it is expected to possess a highly pH-sensitive character. In order to enhance its stability and minimize its pH dependence, we are currently investigating the exchange of at least one of the weak polyelectrolyte blocks (i.e., PAA or PDMAEMA) for a strong one (e.g., PSPMA, quaternized PDMAEMA, or P4VP). In addition, strategies for further improvement of our zipper brush design include the incorporation of a zwitterionic antifouling block, which is currently under investigation.

## Supporting Information Available

Experimental details concerning the polymer synthesis and analysis (^1^H NMR, GPC, and ATR-FTIR) as well as additional DLS spectra, TEM images, AFM images, CA images, QCM-D graphs, and a literature overview (PDF)
