# Supplementary material for: Scalable Fabrication of Reversible Antifouling Block Copolymer Coatings via Adsorption Strategies
Source: ACS Appl Mater Interfaces. 2023 Apr 5;15(15):19682–94. doi: 10.1021/acsami.3c01060 (PMC10119854; doi:10.1021/acsami.3c01060)
Supplement: Supplementary file 1 — am3c01060_si_001.pdf [file am3c01060_si_001.pdf]

Supporting Information

Scalable Fabrication of Reversible Antifouling Block Copolymer Coatings via Adsorption Strategies

Anna M. C. Maan[a], Chantal N. Graafsma[a], Anton H. Hofman[a], Théophile Pelras[b], Wiebe M. de Vos[c*] and Marleen Kamperman[a*]

[a]*Polymer Science, Zernike Institute for Advanced Materials, University of Groningen, Nijenborgh 4, 9747 AG, Groningen, The Netherlands.*

[b]*Macromolecular Chemistry and New Polymeric Materials, Zernike Institute for Advanced Materials, University of Groningen, Nijenborgh 4, 9747 AG, Groningen, The Netherlands.*

[c]*Membrane Science and Technology, MESA+ Institute for Nanotechnology, University of Twente, P.O. Box 217, 7500 AE, Enschede, The Netherlands.*

*Email: w.m.devos@utwente.nl
*Email: marleen.kamperman@rug.nl

1. Polymer Synthesis and Analysis

RAFT synthesis of P*t*BA$_{107}$

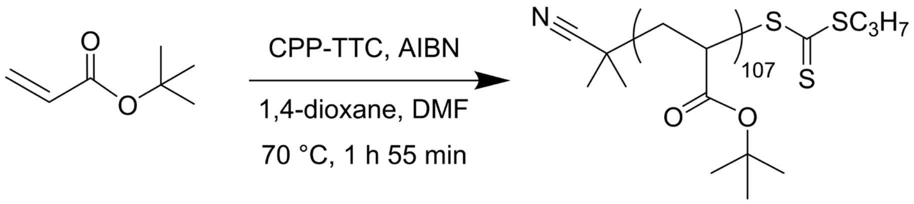

Scheme S1. Reaction scheme for the synthesis of the P*t*BA$_{107}$ homopolymer.

P*t*BA$_{107}$ was synthesized using CPP-TTC as chain transfer agent, as this CTA is less prone to hydrolysis and is more stable towards strong nucleophiles, which is an important factor for the subsequent deprotection step. The chain transfer agent was synthesized according to a previously reported method.[1]

Purified *tert*-butyl acrylate (*t*BA) (200 eq., 91.2 mmol, 11.7 g), CPP-TTC (1 eq., 0.456 mmol, 100 mg), AIBN (0.1 eq., 0.047 mmol, 7.7 mg), 1,4-dioxane (365 mmol, 31 mL) and DMF (200 eq., 91.2 mmol, 6.7 mL) were charged in a 100 mL round-bottom flask equipped with a stirring egg and septum. After complete dissolution, the reaction mixture was sparged with nitrogen for 10 minutes. A t = 0 h ^{1}H NMR sample (~ 2 drops in $CDCl_3$) was taken towards the end of the degassing cycle. The flask was placed in a thermostated oil bath at 70 °C. After 1 h and 55 min, the reaction was quenched by cooling the flask in cold water and subsequently exposing the reaction mixture to air. A t = 2 h ^{1}H NMR sample was prepared (~ 2 drops in $CDCl_3$) in order to calculate the conversion through comparison of the DMF standard and *t*BA peaks (conv. = 53%). The yellow reaction mixture was purified by precipitating into cold methanol / deionized water (3:1, 400 mL). The precipitated product was collected by vacuum filtration and air-dried on the filter. The polymer product was redissolved in 1,4-dioxane and the precipitation procedure was repeated. The yellowish powder was collected in a glass Petri dish to further dry in a vacuum oven (40 °C) overnight. After drying, the yield was determined (5.42 g, 87%) and the product was characterized by ^{1}H NMR (**Figure S1**), GPC (**Figure S2**) and ATR-FTIR (**Figure S4**).

^{1}H NMR (400 MHz, $CDCl_3$): δ (ppm) = 3.31 (t, s-CH_2, CTA), 2.21 (br, CH, backbone), 1.95-1.15 (br, CH_2, backbone), 1.43 (s, -$C(CH_3)_3$). Conversion = 53%, $M_{n,NMR}$ = 13.7 kg mol^{-1}, $P_{n,NMR}$ = 107.

GPC (DMF): $M_{n,GPC}$ = 13.4 kg mol^{-1}, $Đ$ = 1.14.

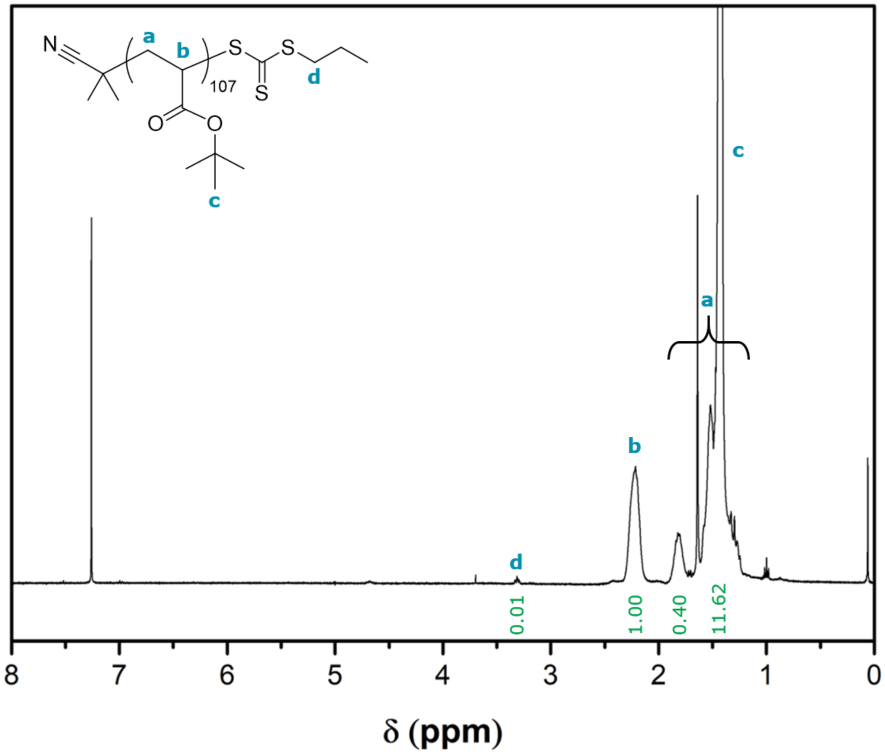

Figure S1. ^{1}H NMR spectrum ($CDCl_3$) of the P*t*BA$_{107}$ homopolymer. $M_{n,NMR}$ = 13.7 kg mol^{-1}, $P_{n,NMR}$ = 107.

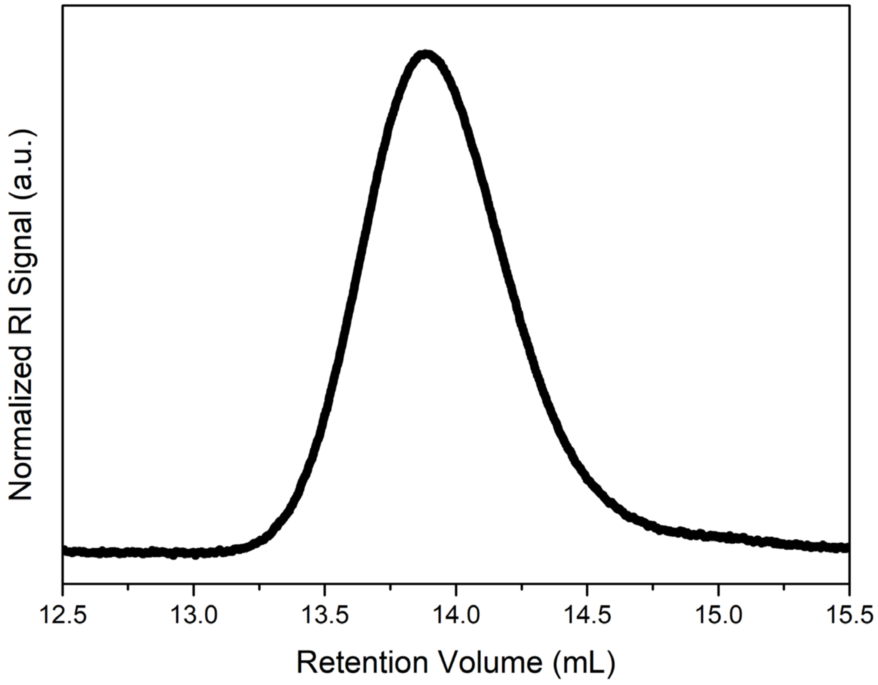

Figure S2. GPC chromatogram (DMF) of the P*t*BA$_{107}$ homopolymer. $M_{n,GPC}$ = 13.4 kg mol^{-1}, $Đ$ = 1.14.

Deprotection of P*t*BA$_{107}$

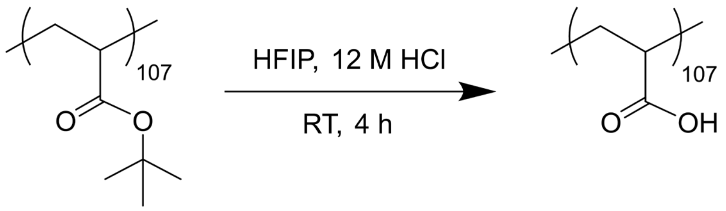

Scheme S2. Reaction scheme for the deprotection of the P*t*BA$_{107}$ homopolymer.

The deprotection of poly(*tert*-butyl acrylate) (P*t*BA) to give poly(acrylic acid) (PAA) was performed according to a previously reported procedure.[2]

P*t*BA$_{107}$ (1 eq., 0.139 mmol, 2.71 g) was charged in a 500 mL round-bottom flask and dissolved in 1,1,1,3,3,3-hexafluoro-2-propanol (HFIP, 170 mL). Once dissolved, 12 M HCl (0.65 eq., 0.181 mmol, 2.3 mL) was added dropwise to the stirred polymer solution. After 4 hours, the solvent was removed in vacuo using a rotary evaporator and the obtained deprotected product was dissolved in ethanol and precipitated twice into *n*-pentane (500 mL). The precipitated product was collected by vacuum filtration and transferred to a glass Petri dish to further dry in a vacuum oven (40 °C) overnight. After drying, the yield was determined (1.30 g, 86%) and the product was characterized by ^{1}H NMR (**Figure S3**) and ATR-FTIR (**Figure S4**).

^{1}H NMR (400 MHz, DMSO-*d*$_6$): δ (ppm) = 12.23 (s, -OH), 2.20 (s, CH, backbone), 1.95-1.15 (br, CH$_2$, backbone).

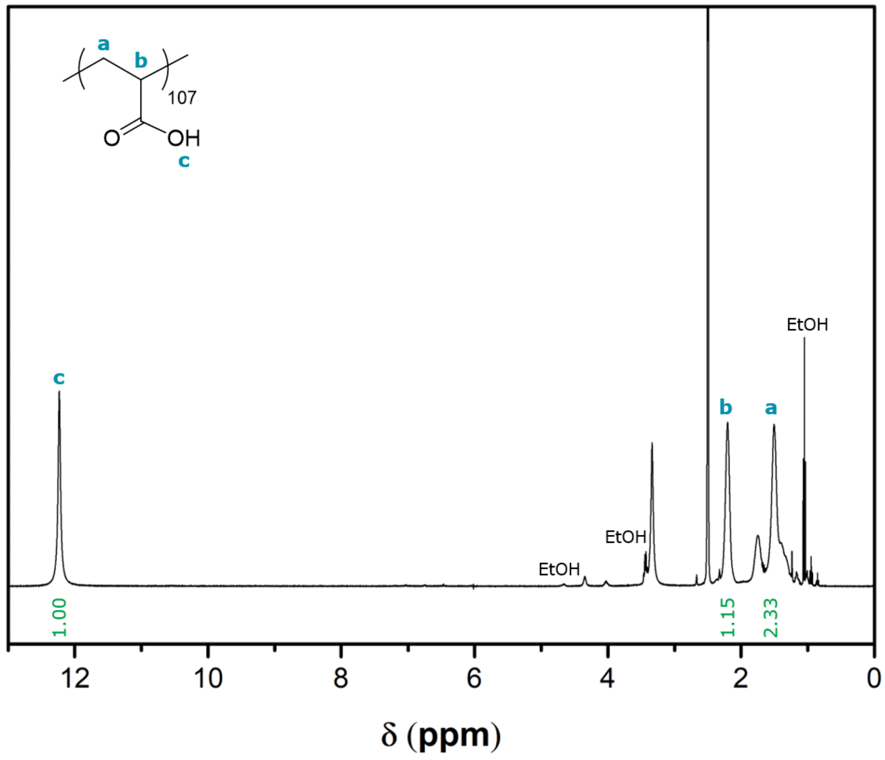

Figure S3. ^{1}H NMR spectrum (DMSO-*d*$_6$) of the PAA$_{107}$ homopolymer.

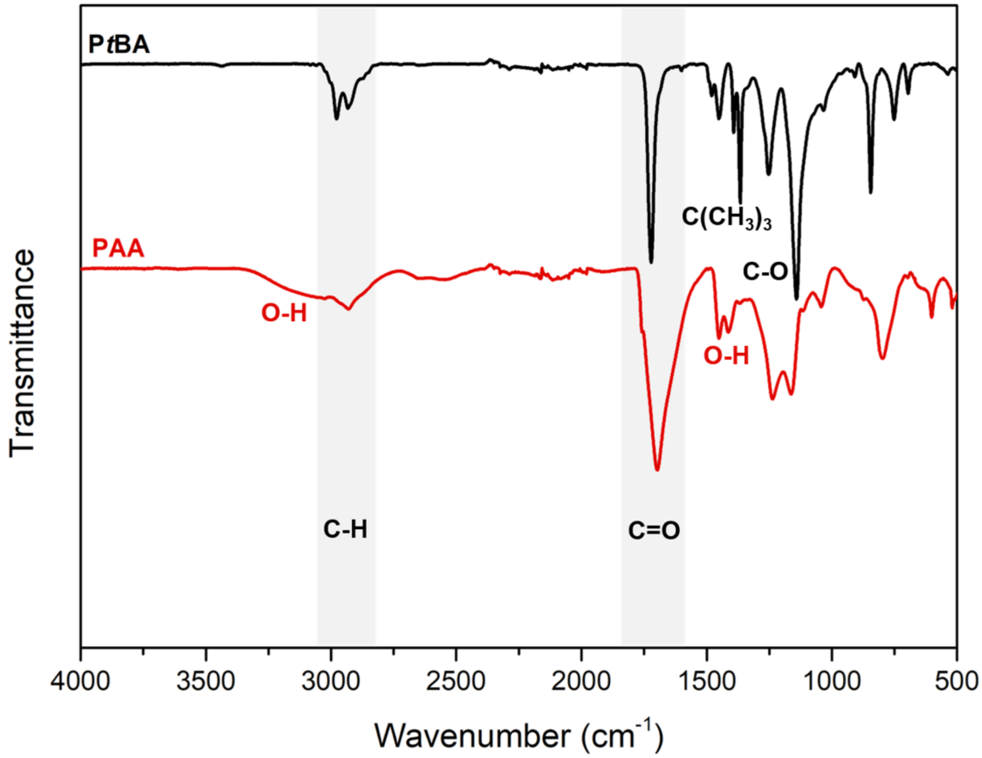

Figure S4. ATR-FTIR spectra of the synthesized P*t*BA$_{107}$ (black) and PAA$_{107}$ (red) homopolymers. Comparison of the spectra confirms the successful deprotection of P*t*BA to give PAA, marked by the absence of the -C(CH$_3$)$_3$ (1366 cm^{-1}) and C-O (1141 cm^{-1}) stretching vibrations, as well as the broadening of the adsorption bands around 3000 cm^{-1} (-OH stretch) and 1724 cm^{-1} (C=O stretch), corresponding to the carboxylic acid group.

Bulk synthesis of the PS_{81} macro-CTA by RAFT polymerization

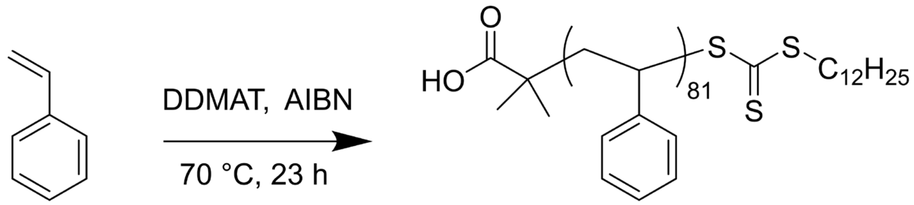

Scheme S3. Reaction scheme for the synthesis of the PS_{81} macro-CTA.

The polystyrene macro-CTA was synthesized according to an adapted literature procedure.[3] Purified styrene (135 eq., 36.7 mmol, 3.82 g, 4.20 mL), DDMAT (1 eq., 0.273 mmol, 99.6 mg) and AIBN (0.11 eq., 0.031 mmol, 5.1 mg) were charged in a 25 mL round-bottom flask, equipped with a stirring egg and a septum. Once all chemicals were dissolved, the yellow reaction mixture was sparged with nitrogen for 10 minutes. The flask was placed in a thermostated oil bath at 70 °C while stirring continuously. After 23 h, the reaction was quenched by cooling the flask in cold water and subsequently exposing the reaction mixture to air. A t = 23 h ^{1}H NMR sample was prepared (~ 2 drops in $CDCl_3$) in order to calculate the conversion through comparison of the polymer and monomer peaks (conv. = 60.3%). The viscous yellow polymer mixture was purified by precipitating twice from methanol: the polymer mixture was first diluted with a small amount of THF (~ 2 mL) and precipitated dropwise into a beaker containing thoroughly stirred methanol (500 mL). The precipitated product was collected by vacuum filtration, washed with methanol and air-dried on the filter for at least one hour. The polymer product was redissolved in THF and the precipitation procedure was repeated. The precipitated yellow powder was collected in a Petri dish and dried in a vacuum oven (40 °C) overnight. After drying, the yield was determined (1.23 g, 52%) and the product was characterized by ^{1}H NMR (**Figure S5**), GPC (**Figure S7**) and ATR-FTIR (**Figure S11**).

^{1}H NMR (400 MHz, $CDCl_3$): δ (ppm) = 7.30-6.30 (br, 5 H, aromatic ring), 3.26 (br, S-CH_2, CTA), 2.30-1.70 (br, CH, backbone), 1.70-1.30 (br, CH_2, backbone), 1.27 (br, $C_{10}H_{20}$, CTA), 0.89 (br, CH_3, CTA). Conversion = 60.3%, $M_{n,PS}$ = 8.80 kg mol^{-1}, $P_{n,PS}$ = 81. According to end-group analysis based on DDMAT and the aromatic ring: $P_{n,PS}$ = 79.

GPC (DMF): $M_{n,GPC}$ = 8.12 kg mol^{-1}, $Đ$ = 1.09.

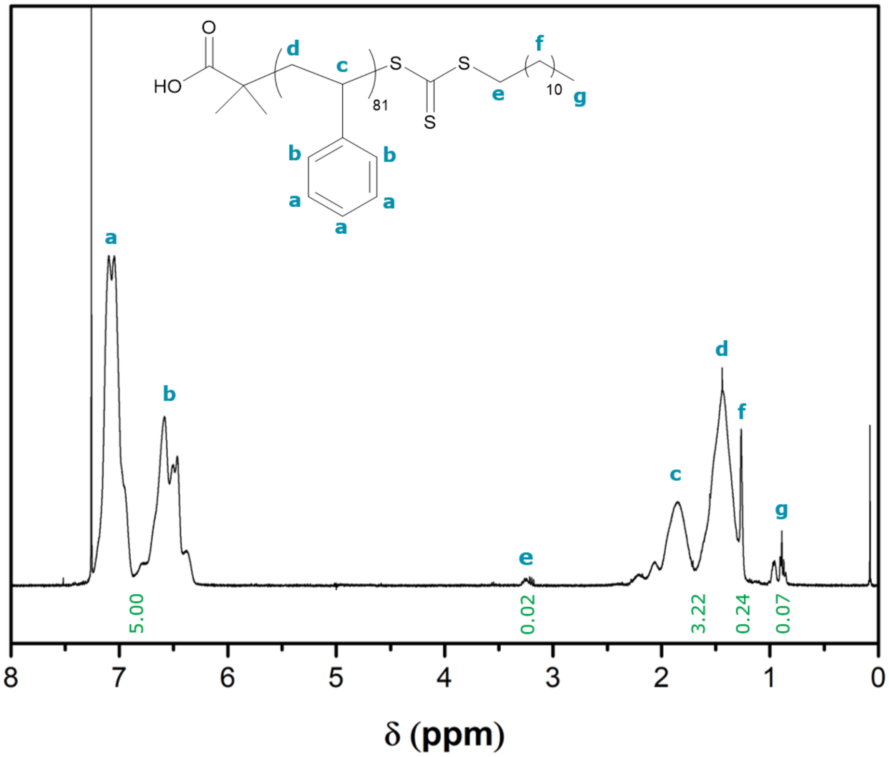

Figure S5. ^{1}H NMR spectrum ($CDCl_3$) of the PS_{81} macro-CTA. $M_{n,NMR}$ = 8.80 kg mol^{-1}, $P_{n,NMR}$ = 81.

RAFT synthesis of PS_{81}-*b*-P*t*BA_{81}

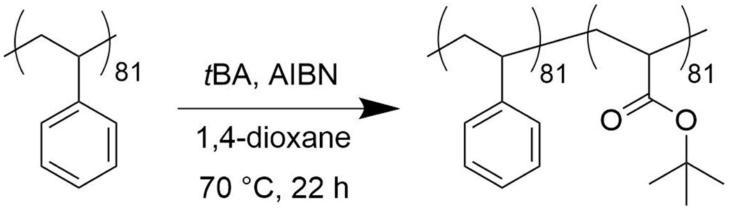

Scheme S4. Reaction scheme for the synthesis of the PS_{81}-*b*-P*t*BA_{81} diblock copolymer.

Purified *t*BA (83.5 eq., 8.55 mmol, 1.10 g, 1.25 mL), PS_{81}-CTA (1 eq., 0.102 mmol, 900 mg), AIBN (0.11 eq., 0.012 mmol, 1.90 mg - using a 1 mg mL^{-1} AIBN stock solution in 1,4-dioxane), and 1,4-dioxane (1 mL) were charged in a 20 mL glass vial and mixed until everything was dissolved. After complete dissolution, the yellow mixture was carefully transferred to a 25 mL round-bottom flask, equipped with a stirring egg and a septum. The reaction mixture was sparged with nitrogen for 10 minutes. A t = 0 h ^{1}H NMR sample (~ 2 drops in $CDCl_3$) was taken towards the end of the degassing cycle. The flask was placed inside a thermostated oil bath at 70 °C. After stirring for 22 h, the reaction was quenched by cooling the flask in cold water and subsequently exposing the reaction mixture to air. A t = 22 h ^{1}H NMR sample was prepared (~ 2 drops in $CDCl_3$) in order to calculate the conversion through comparison of the polystyrene and *t*BA peaks (conv. = 98.8%). The viscous yellow polymer mixture was purified by precipitating the undiluted solution into thoroughly stirred methanol (500 mL). The precipitated product was collected by vacuum filtration, washed with methanol and air-dried on the filter for several minutes. The yellowish powder was collected in a Petri dish and dried in a vacuum oven (40 °C) overnight. After drying, the yield was determined (1.12 g, 56%) and the product was characterized by ^{1}H NMR (**Figure S6**), GPC (**Figure S7**) and ATR-FTIR (**Figure S11**).

^{1}H NMR (400 MHz, $CDCl_3$): 7.30-6.30 (br, 5 CH, PS aromatic ring), 3.33 (br, S-CH_2, CTA), 2.35-1.10 (br, CH and CH_2, backbone PS and P*t*BA), 1.44 (s, -$C(CH_3)_3$, P*t*BA), 1.26 (br, $C_{10}H_{20}$, CTA), 0.90 (br, CH_3, CTA). Conversion = 98.8%, $M_{n,PtBA}$ = 10.4 kg, $P_{n,PtBA}$ = 81. $M_{n,total}$ = 19.2 kg mol^{-1}. x_{PtBA} = 0.495, f_{PtBA} = 0.547.

GPC (DMF): $M_{n,GPC}$ = 15.6 kg mol^{-1}, $Đ$ = 1.15.

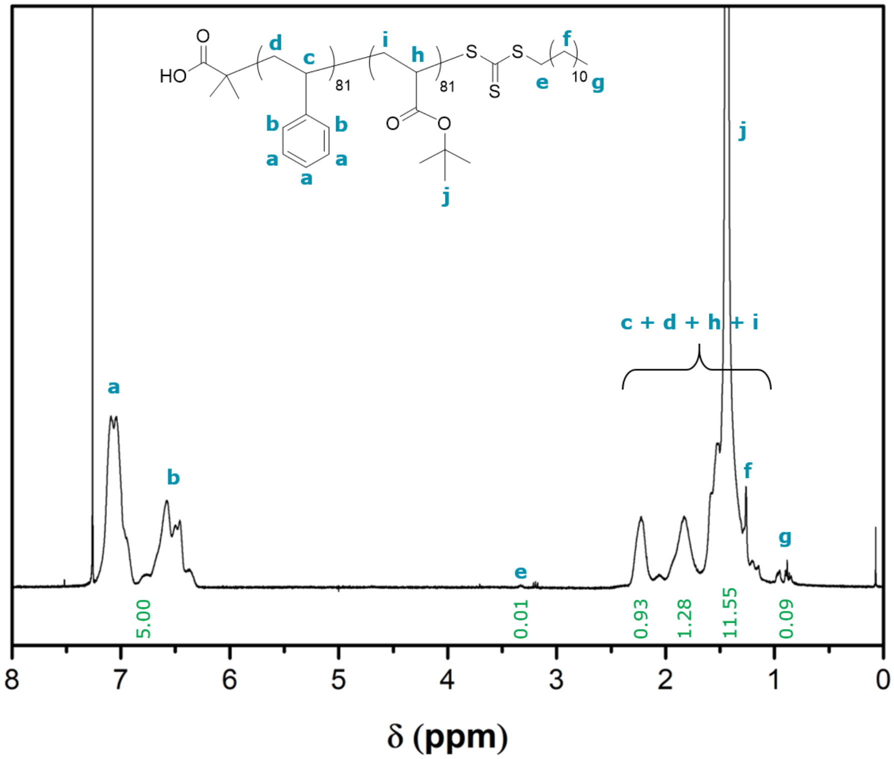

Figure S6. ^{1}H NMR spectrum ($CDCl_3$) of the PS_{81}-*b*-P*t*BA_{81} diblock copolymer. $M_{n,\mathrm{P}t\mathrm{BA}}$ = 10.4 kg mol^{-1}, $P_{n,\mathrm{P}t\mathrm{BA}}$ = 81. $M_{n,\mathrm{total}}$ = 19.2 kg mol^{-1}. x_{PtBA} = 0.495, f_{PtBA} = 0.547.

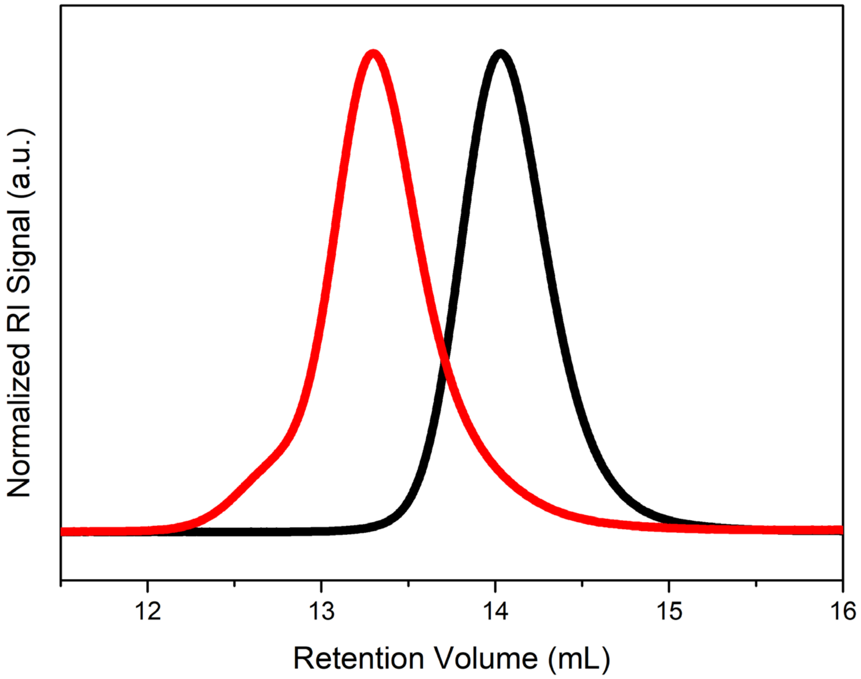

Figure S7. GPC chromatograms (DMF) of the PS_{81} macro-CTA (black) and the PS_{81}-*b*-P*t*BA_{81} diblock copolymer (red). GPC characteristics of PS_{81}-CTA: M_n = 8.12 kg mol^{-1}, $Đ$ = 1.09. GPC characteristics of PS_{81}-*b*-P*t*BA_{81} (red): M_n = 15.6 kg mol^{-1}, $Đ$ = 1.15.

Deprotection of PS_{81}-*b*-P*t*BA_{81}

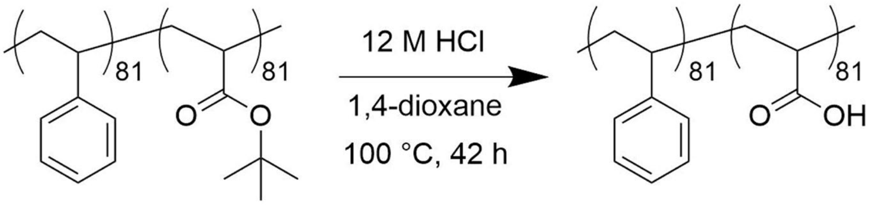

Scheme S5. Reaction scheme for the deprotection of the PS_{81}-*b*-P*t*BA_{81} diblock copolymer.

Due to insolubility of PS in HFIP, a different deprotection method was followed for the PS_{81}-*b*-P*t*BA_{81} diblock copolymer.[4,5]

PS_{81}-*b*-P*t*BA_{81} (0.415g, f_{PtBA} = 0.55, 1 eq. *t*BA, 1.79 mmol *t*BA, 0.23 g *t*BA) was dissolved in 1,4-dioxane (5 mL) in a 50 mL round-bottom flask equipped with a stirring egg and reflux condenser. After dissolution, 12 M HCl (5 eq., 8.98 mmol, 0.86 g, 0.75 mL) was added to the stirred solution and the mixture was heated to 100 °C. After 42 hours, the mixture was cooled down slowly, the solvent was removed in vacuo using a rotary evaporator and a ^{1}H NMR (in DMSO-d_6) was recorded to confirm the successful deprotection. The deprotected polymer was redissolved in 1,4-dioxane (4 mL) and precipitated into *n*-pentane (500 mL). The precipitated light brown powder was collected by vacuum filtration and transferred to a Petri dish to further dry in a vacuum oven (40 °C) overnight. After drying, the yield was determined (0.28 g, 88%) and the product was characterized by ^{1}H NMR (**Figure S8**) and ATR-FTIR (**Figure S11**). The trithiocarbonate end-groups were not affected by the employed deprotection conditions, as was evidenced by ^{1}H NMR and GPC (**Figure S9 and S10**).

^{1}H NMR (400 MHz, DMSO-d_6): δ (ppm) = 12.22 (s, -OH, PAA), 7.40-6.10 (br, 5 CH, PS aromatic ring), 2.42-1.00 (br, CH and CH_2, backbone PS and PAA).

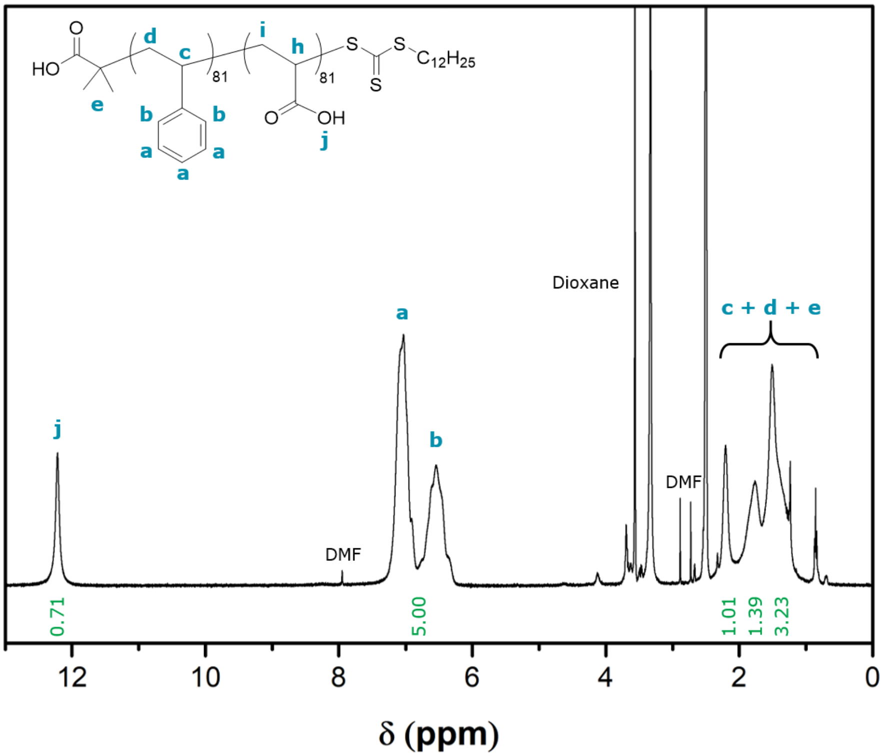

Figure S8. ^{1}H NMR spectrum (DMSO-d_6) of the PS_{81}-*b*-PAA_{81} diblock copolymer.

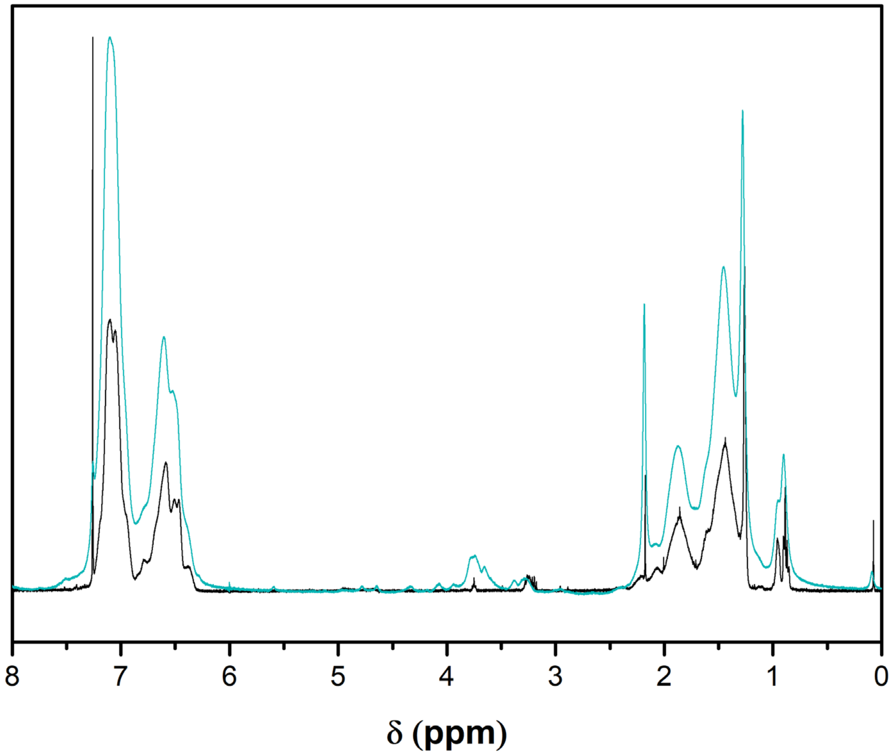

Figure S9. ^{1}H NMR spectra ($CDCl_3$) of the control experiment: PS_{32} homopolymer before (black) and after (blue) performing the 42 h dioxane/HCl reflux deprotection protocol. The peaks representing the RAFT end-groups (3.33, 1.26, 0.90 ppm) are still present after the deprotection protocol, albeit less well-defined (broader signals).

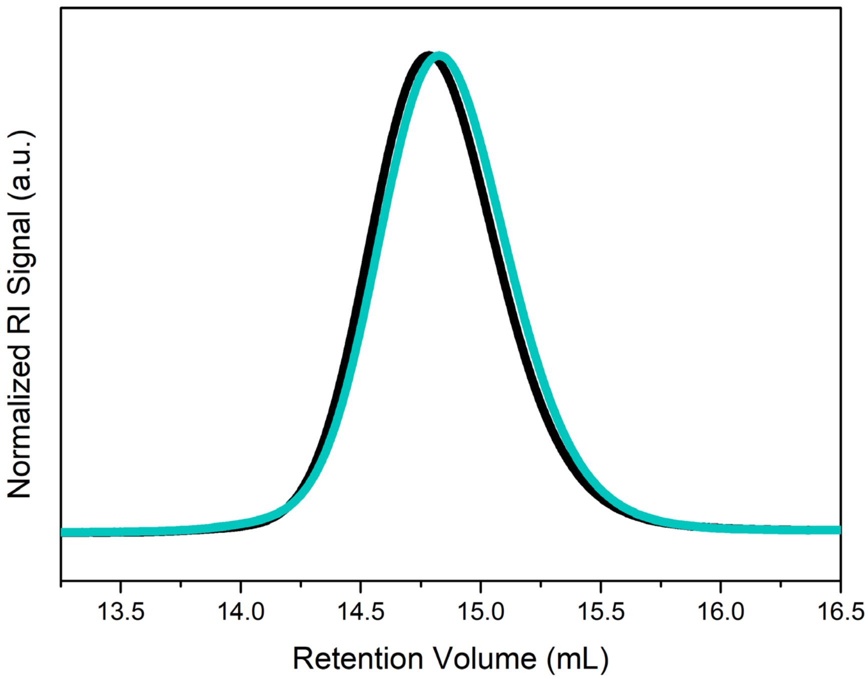

Figure S10. GPC chromatograms (DMF) of the control experiment: PS_{32} homopolymer before (black) and after (blue) performing the 42 h dioxane/HCl reflux deprotection protocol. The single non-shifted peak after deprotection confirms the hypothesis that the RAFT end-groups are stable throughout this process, as there are no signs of chain coupling (i.e., peak doubling).

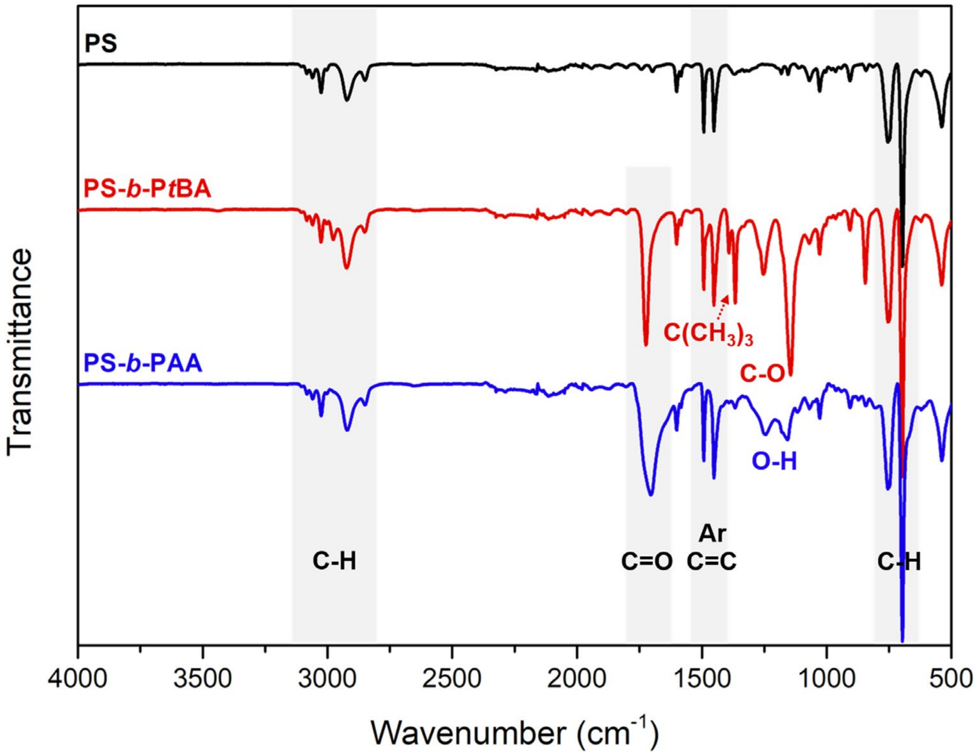

Figure S11. ATR-FTIR spectra of the synthesized PS_{81} macro-CTA (black) and the PS_{81}-*b*-P*t*BA_{81} (red) and PS_{81}-*b*-PAA_{81} (blue) diblock copolymers. Comparison of the spectra confirms the successful addition of the P*t*BA block, evidenced by the emerging characteristic adsorption bands of P*t*BA: the -$C(CH_3)_3$ stretch at 1366 cm^{-1} and the strong C=O (1724 cm^{-1}) and C-O (1144 cm^{-1}) stretching vibrations. In addition, the FTIR spectrum of PS_{81}-*b*-PAA_{81} confirms a successful deprotection, marked by the absence of the -$C(CH_3)_3$ and C-O stretching vibrations, as well as the broadening of the C=O stretching vibration (1724 cm^{-1}).

ATRP synthesis of PEG_{90}-*b*-$PDMAEMA_{29}$

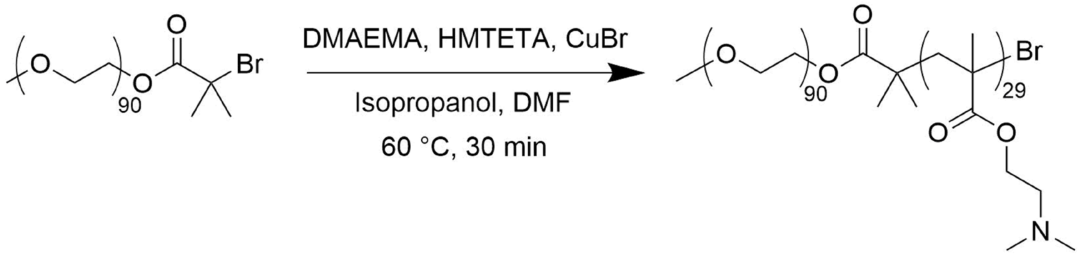

Scheme S6. Reaction scheme for the synthesis of the PEG_{90}-*b*-$PDMAEMA_{29}$ diblock copolymer.

PEG_{90} homopolymer (M_n = 4.01 kg mol^{-1}, $Đ$ = 1.05) was purchased from TCI and subsequently converted into a PEG_{90}-Br macroinitiator (M_n = 4.14 kg mol^{-1}, $Đ$ = 1.06) according to a previously reported procedure.[6] PEG_{90}-Br (1 eq., 0.240 mmol, 995 mg) and isopropanol (560 eq., 7.8 mL) were charged into a 20 mL glass vial. Careful heating of the mixture inside a warm water bath was required to ensure full dissolution of the PEG_{90}-Br macroinitiator. Purified DMAEMA (50 eq., 12.1 mmol, 1.89 g), HMTETA (1 eq., 0.238 mmol, 55.2 mg) and DMF (50 eq., 12.1 mmol, 0.94 mL) were subsequently added to the glass vial and everything was mixed until dissolved. The transparent reaction mixture was transferred to a 100 mL Schlenk flask, equipped with a stirring egg and septum, and a t = 0 h ^{1}H NMR sample (~ 2 drops in $CDCl_3$) was taken. The reaction mixture was deoxygenated via three freeze-pump-thaw cycles, after which the reaction mixture was frozen once more and the CuBr catalyst powder (1 eq., 0.239 mmol, 34.0 mg) was carefully added on top of the frozen mixture with the Ar/vacuum inlet closed. Afterwards, the flask was closed with a septum and the freeze-pump-thaw cycle was continued. During the final thaw step, the septum was punctured with a short needle to flush the flask with argon in order to remove any remaining oxygen. Once fully defrosted, the degassing cycle was stopped (removal of outlet needle, closing of inlet valve), and the flask was placed inside a thermostated oil bath at 60 °C to start the reaction. After 30 minutes, the green reaction mixture was quenched by cooling the flask in cold water and subsequently exposing the reaction mixture to air. A t = 30 min ^{1}H NMR sample was prepared (~ 2 drops in $CDCl_3$) in order to calculate the conversion through comparison of the DMF standard and DMAEMA peaks (conv. = 59 %). The reaction mixture was precipitated once in *n*-hexane, redissolved in THF and passed through a short Al_2O_3 (basic) column to remove excess CuBr catalyst. The polymer solution was transferred to a dialysis membrane (Spectra/Por 6, Spectrum Laboratories, 1 kDa molecular weight cut-off) and dialysis was performed against methanol over the course of several days (≥ 3 days), while frequently replenishing the solvent. The polymer solution was concentrated in vacuo using a rotary evaporator and further dried in a vacuum oven (40 °C) overnight. After drying, the yield was determined (1.40 g, 66%) and the product was characterized by ^{1}H NMR (**Figure S12**), GPC (**Figure S13**) and ATR-FTIR (**Figure S14**).

^{1}H NMR (400 MHz, $CDCl_3$): δ (ppm) = 4.05 (br, O-CH_2, PDMAEMA), 3.63 (s, 2x CH_2, PEG), 2.55 (br, CH_2-N, PDMAEMA), 2.27 (s, 2x N-CH_3, PDMAEMA), 2.05-1.70 (br, CH_2 backbone, PDMAEMA), 1.16-0.75 (br, C-CH_3, PDMAEMA). Conversion = 59%, $M_{n,PDMAEMA}$ = 4.56 kg mol^{-1}, $P_{n,NMR}$ = 29. $M_{n,total}$ = 8.70 kg mol^{-1}.

GPC (DMF): $M_{n,GPC}$ = 13.8 kg mol^{-1}, $Đ$ = 1.22.

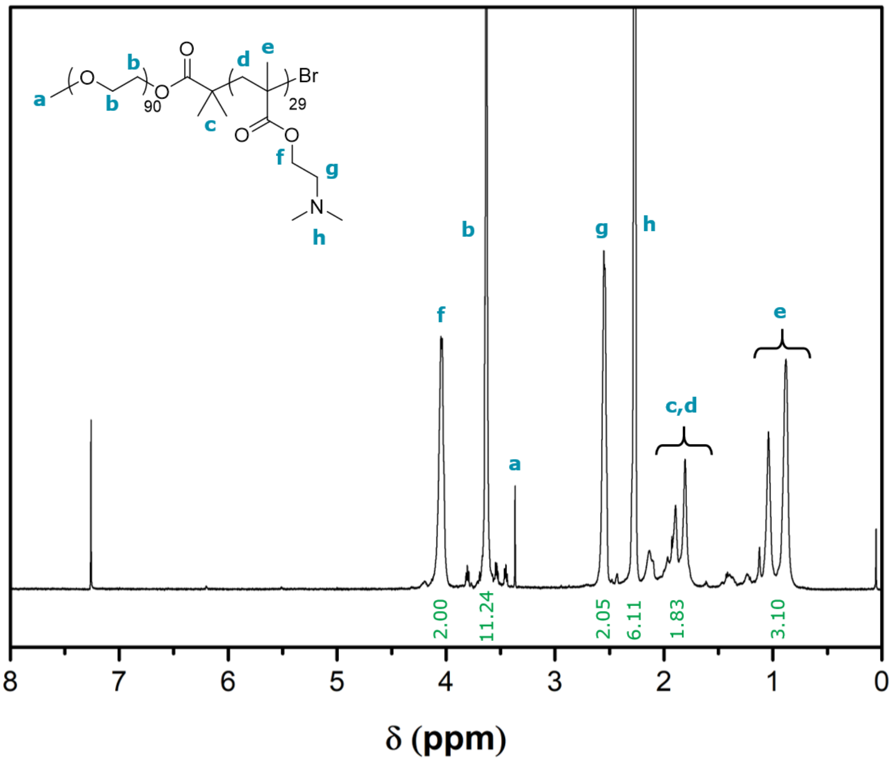

Figure S12. ^{1}H NMR spectrum ($CDCl_3$) of the PEG_{90}-*b*-$PDMAEMA_{29}$ diblock copolymer. $M_{n,PDMAEMA}$ = 4.56 kg mol^{-1}, $P_{n,NMR}$ = 29. $M_{n,total}$ = 8.70 kg mol^{-1}.

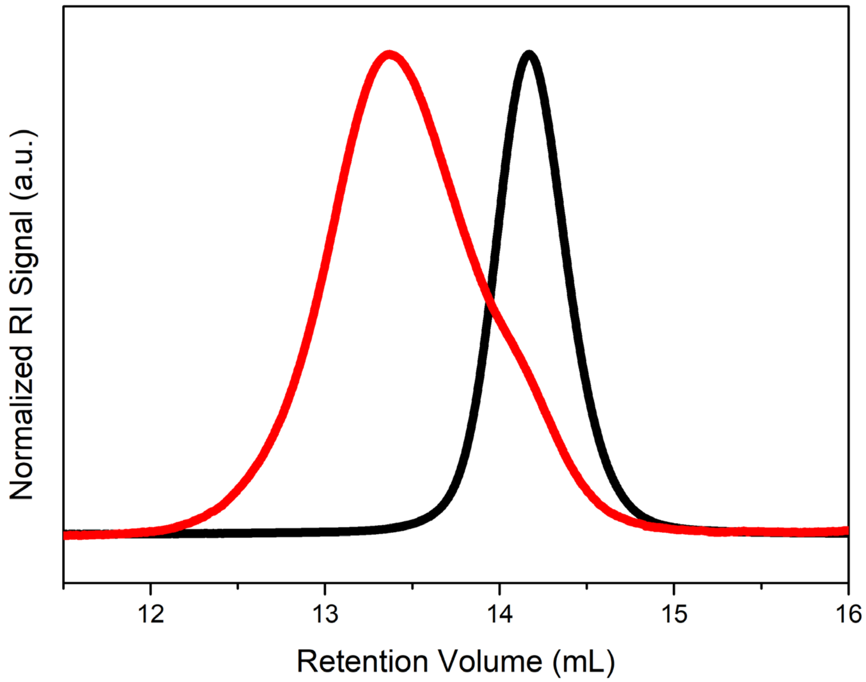

Figure S13. GPC chromatograms (DMF) of the PEG_{90}-Br macroinitiator (black) and PEG_{90}-*b*-$PDMAEMA_{29}$ diblock copolymer (red). GPC characteristics of PEG_{90}-Br: M_n = 7.04 kg mol^{-1}, $Đ$ = 1.06. GPC characteristics of PEG_{90}-*b*-$PDMAEMA_{29}$ (red): M_n = 13.8 kg mol^{-1}, $Đ$ = 1.22.

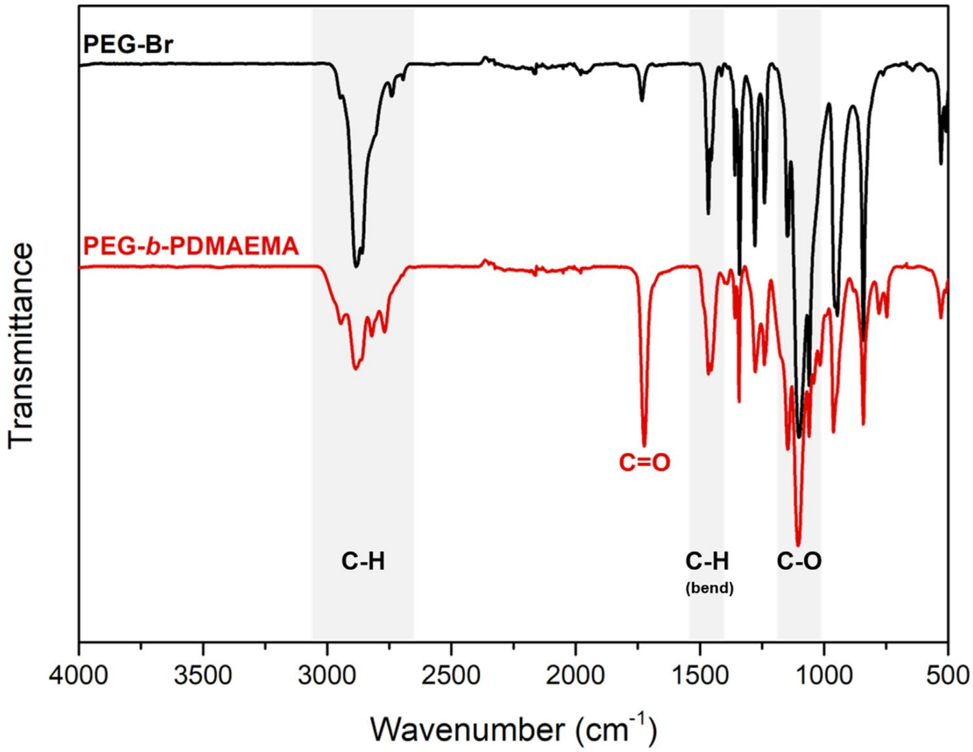

Figure S14. ATR-FTIR spectra of the PEG_{90}-Br macroinitiator (black) and PEG_{90}-*b*-$PDMAEMA_{29}$ diblock copolymer (red). The strong C=O stretch emerging at 1724 cm^{-1} and the broadened C-H stretching vibration confirm the successful addition of the PDMAEMA block.

2. Results and Discussion - Extra Analysis Data

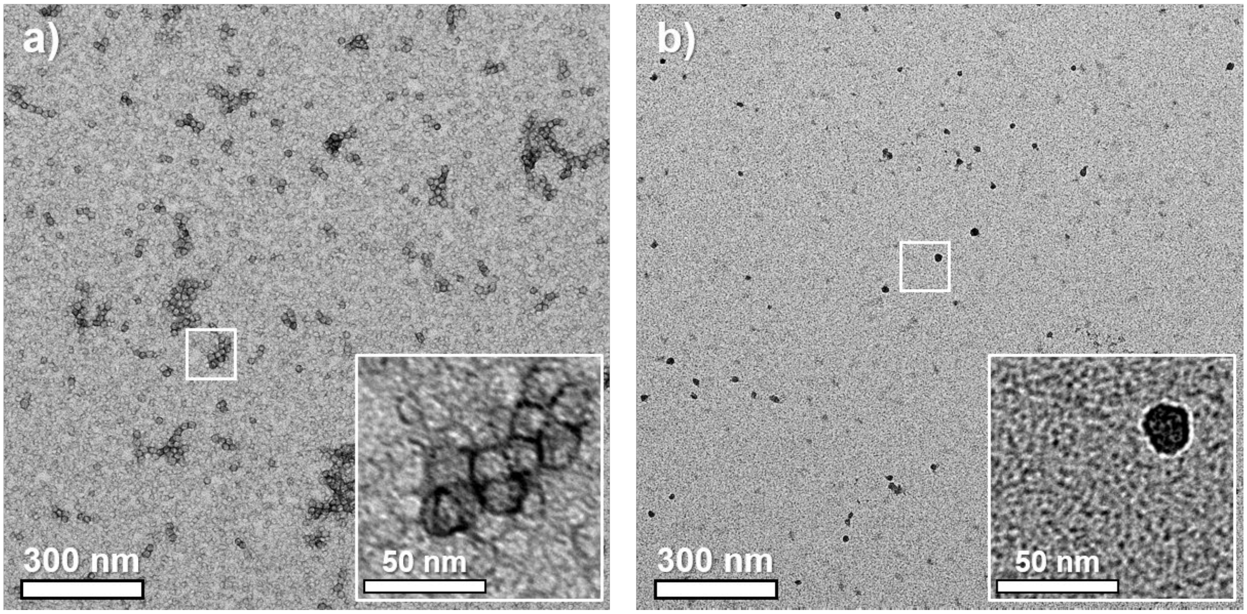

Figure S15. TEM images of uranyl acetate-stained (a) PS-*b*-PAA micelles and (b) C3Ms. The average diameter (± SD) of the PS-*b*-PAA micelles is 15.0 ± 1.6 nm, while the C3Ms have a diameter of 16.3 ± 2.4 nm. The recorded dimensions measured in TEM are smaller than the values found with DLS, which can be attributed to drying and/or staining effects. It is interesting to note that the stain accumulates on the outer parts of the PS-*b*-PAA micelles (i.e., negative staining), while it penetrates into the C3M core (i.e., positive staining), a phenomenon we have observed before.[6] Hence, the diameter indicated for the C3Ms is classified as the core diameter, as it is not taking into account the PEG corona.

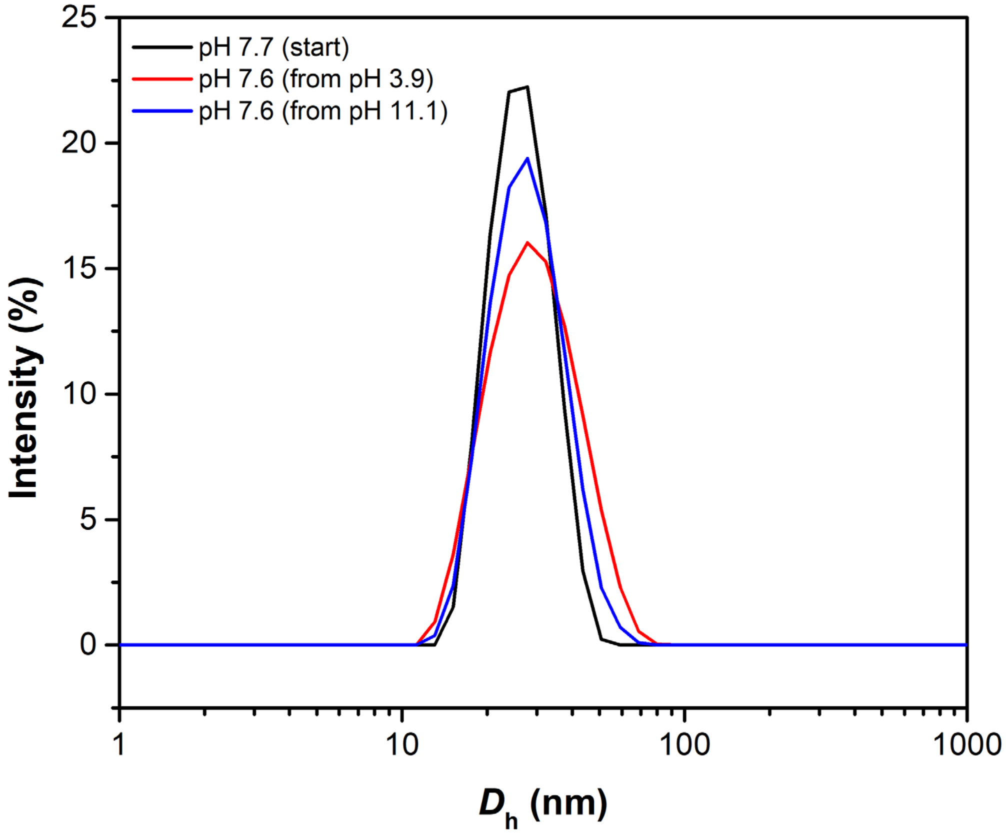

Figure S16. Size distribution plots of the C3Ms before and after pH cycling. C3Ms dissociate outside the optimum pH regime of pH = 6.5 – 8.4, but readily reform once the pH is adjusted back to the stable range.

**pH**	**Restored from pH**	D_h (nm)	**PDI** (nm)	ζ (mV)
7.7	-	28.4	0.21	-0.8
7.6	3.9	26.6	0.14	+2.9
7.6	11.1	28.0	0.19	+1.0

Table S1. Hydrodynamic diameters (D_h), polydispersity indices (PDI) and zeta potentials (ζ) of the initial and reformed C3Ms.

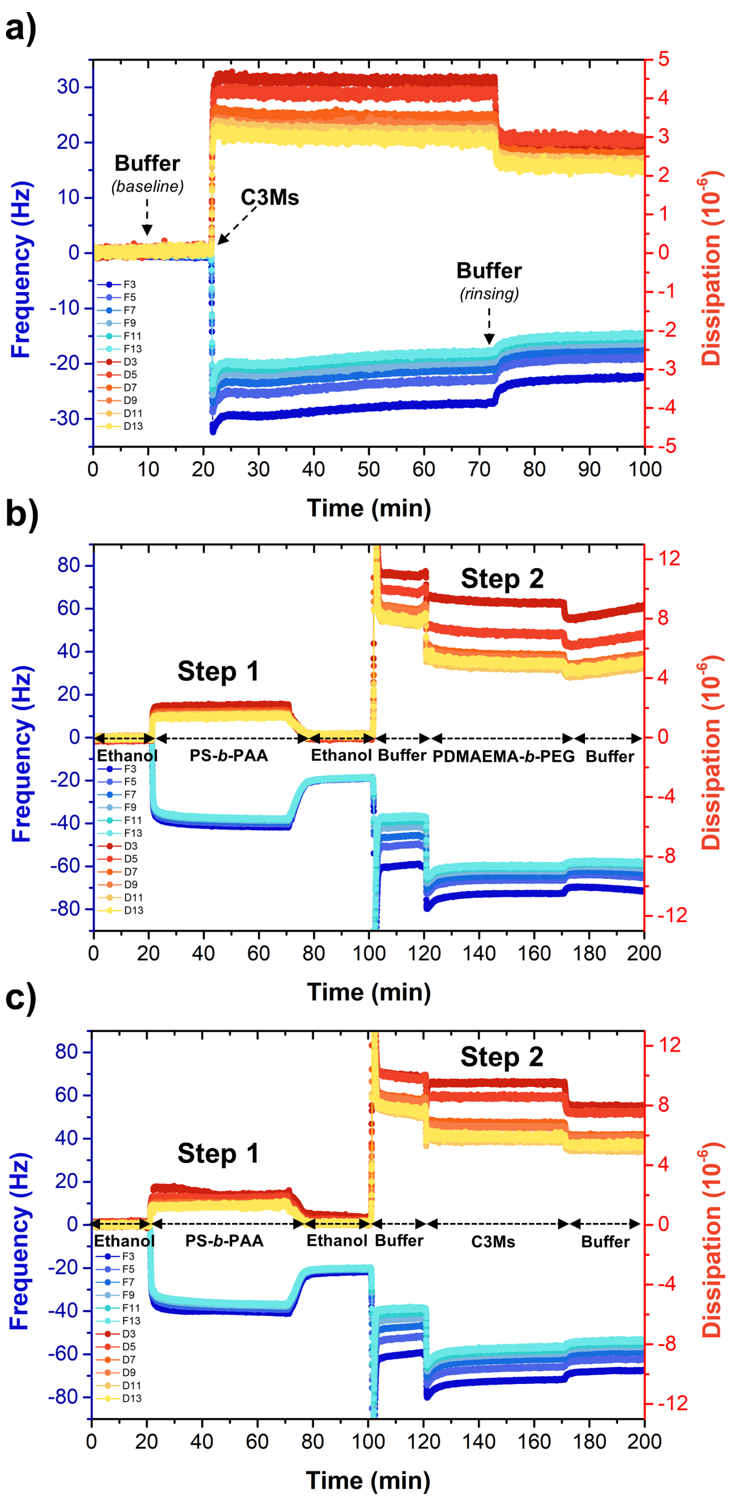

Figure S17. QCM-D graphs showing the in situ formation of the (a) C3M coating, (b) zipper brush and (c) hybrid coating, including all harmonic overtones (R3-R13). The total shifts in both frequency (F3-F13, blue) and energy dissipation (D3-D13, red) are depicted. Due to insufficient energy trapping, the Δ*f* and Δ*D* values from the fundamental frequency (F1, D1) were usually noisy and were therefore excluded from further analysis.

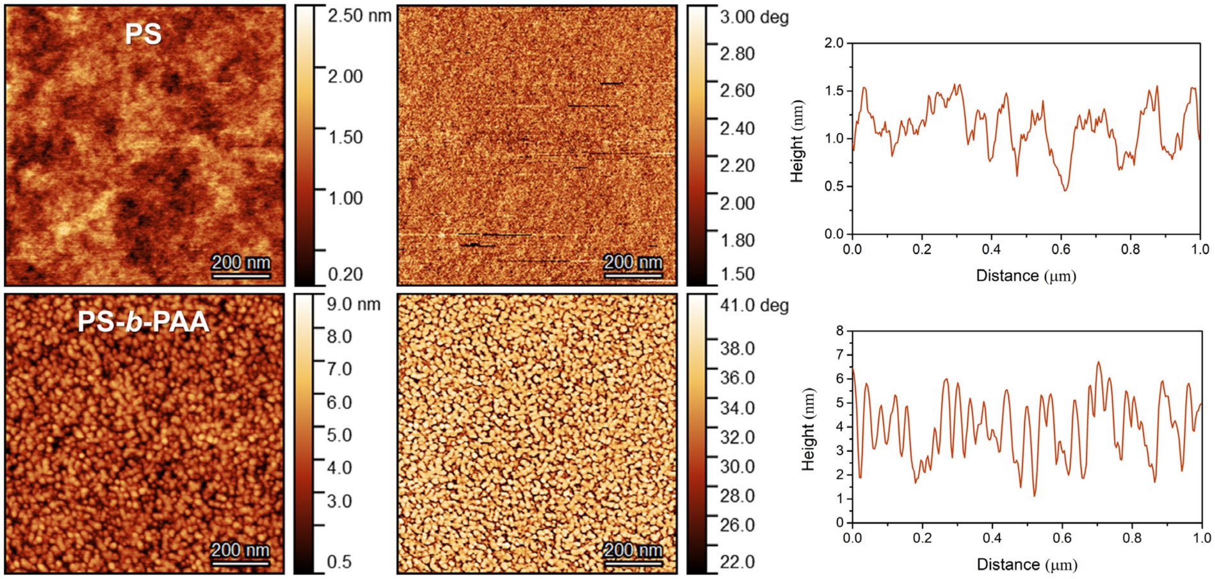

Figure S18. AFM height images (left) and corresponding phase images (middle) and cross-sectional profiles (right) of the spin-coated PS thin film and adsorbed PS-*b*-PAA primer.

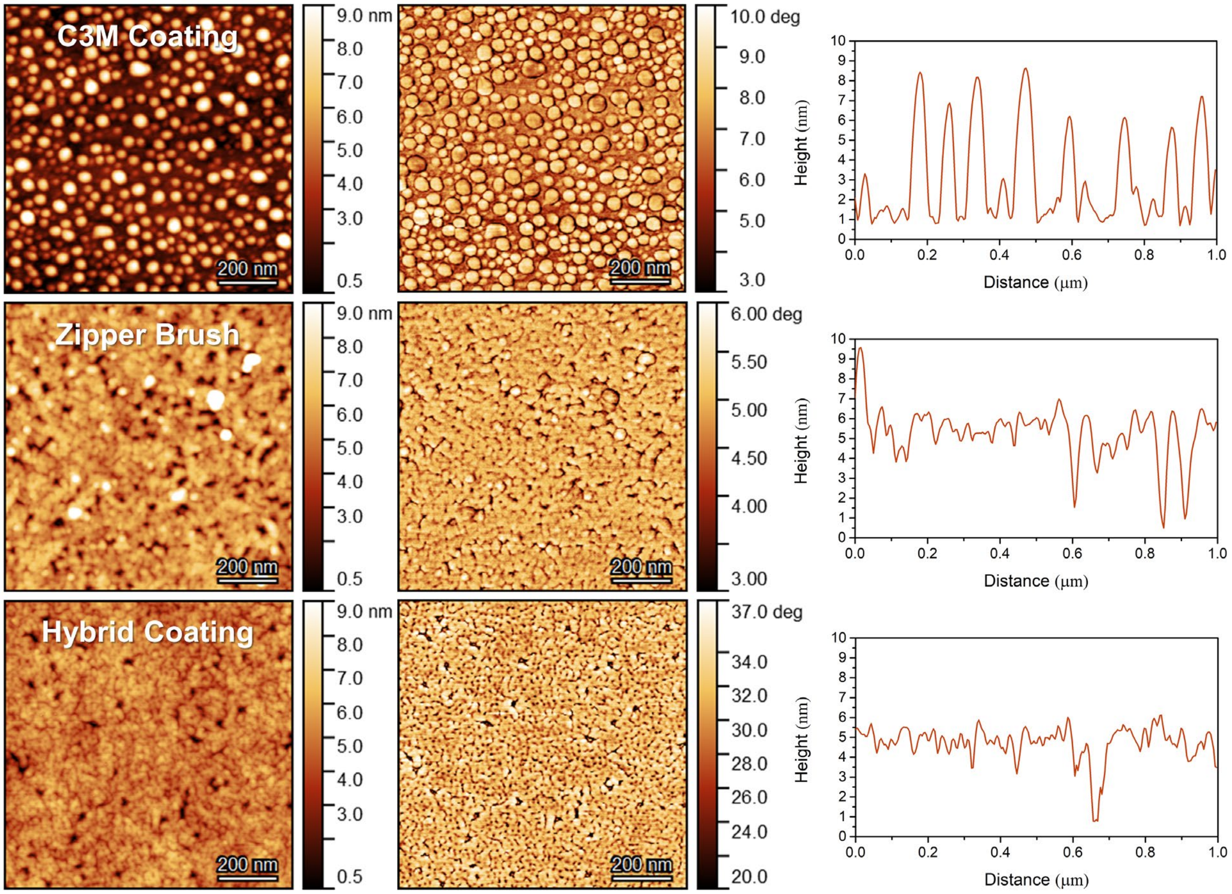

Figure S19. AFM height images (left) and corresponding phase images (middle) and cross-sectional profiles (right) of the three antifouling polymer-based coatings.

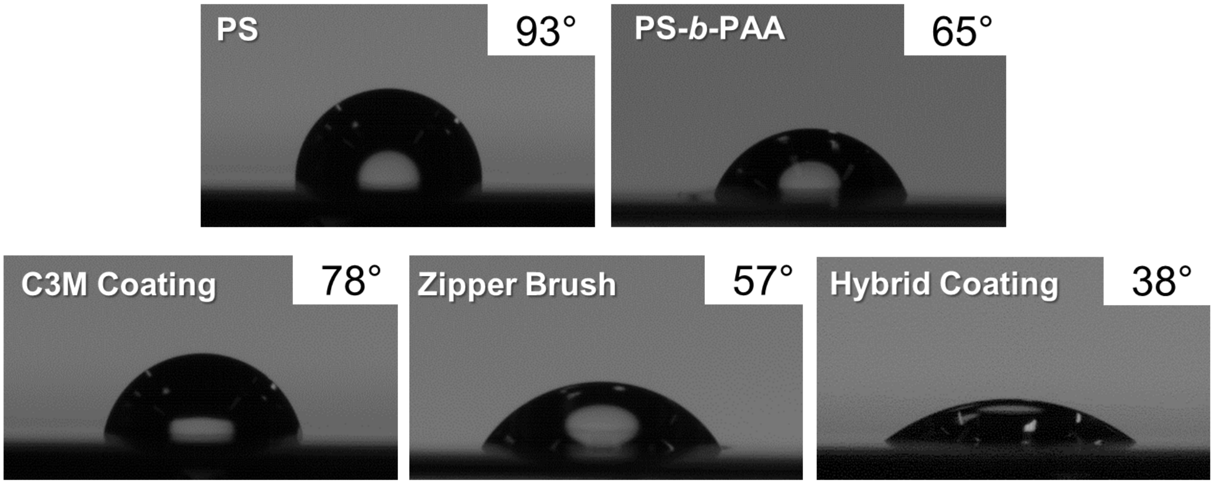

Figure S20. Static contact angles of all coated surfaces, representing the intrinsic water wettability.

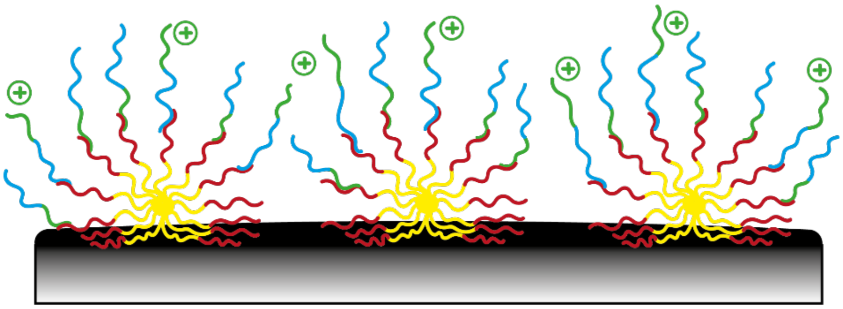

Figure S21. Schematic representation of the zipper brush morphology lacking the desired conformation: the PEG blocks may also interact with the pre-adsorbed PAA chains via hydrogen bonds, thereby positioning the positively charged PDMAEMA chains towards the surface, resulting in a net positive surface charge. This conformation will facilitate the adhesion of negatively charged BSA.

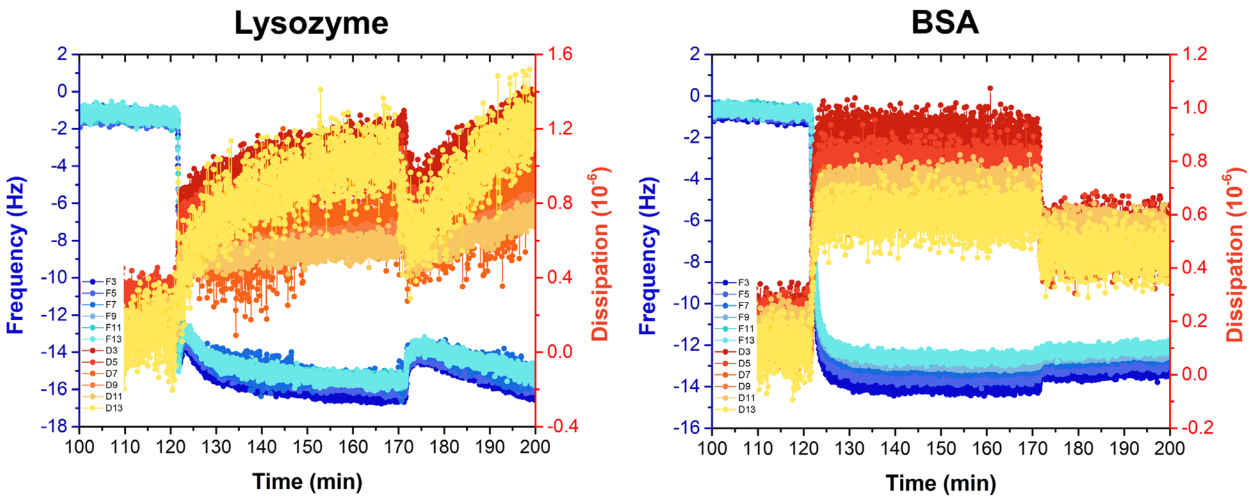

Figure S22. QCM-D graphs representing the antifouling performance of the pristine PS-coated surface against lysozyme and BSA. The pronounced negative frequency shifts indicate that both lysozyme and BSA adsorb to PS, and they do so with a high adhesion strength: rinsing with buffer (at 170 min) barely removes any material.

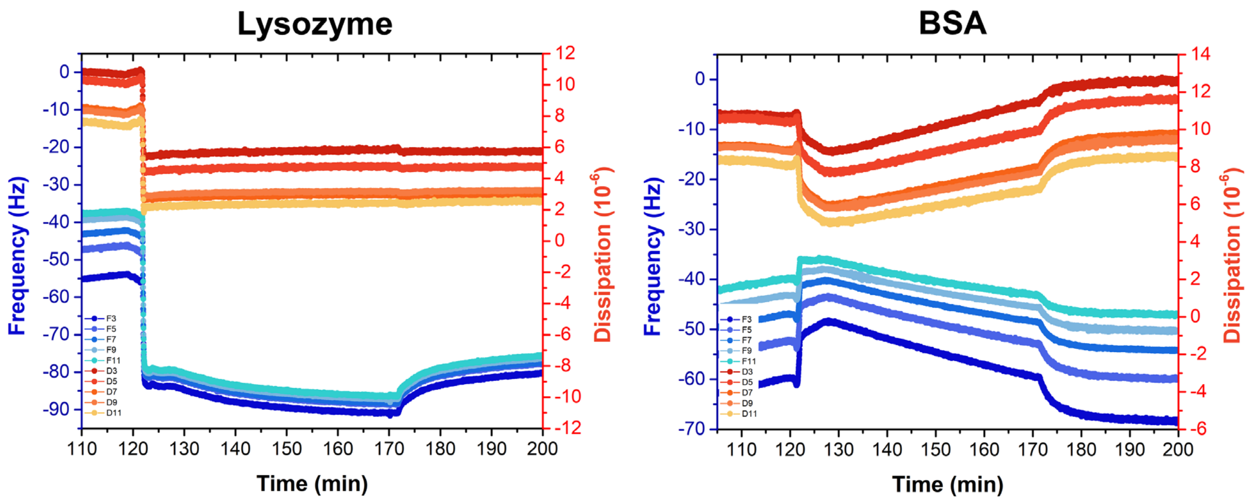

Figure S23. QCM-D graphs representing the antifouling performance of the negatively charged PS-*b*-PAA primer against lysozyme and BSA. While positively charged lysozyme readily adheres to this primer layer, negatively charged BSA seems to be mostly repelled, as was expected based on electrostatics.

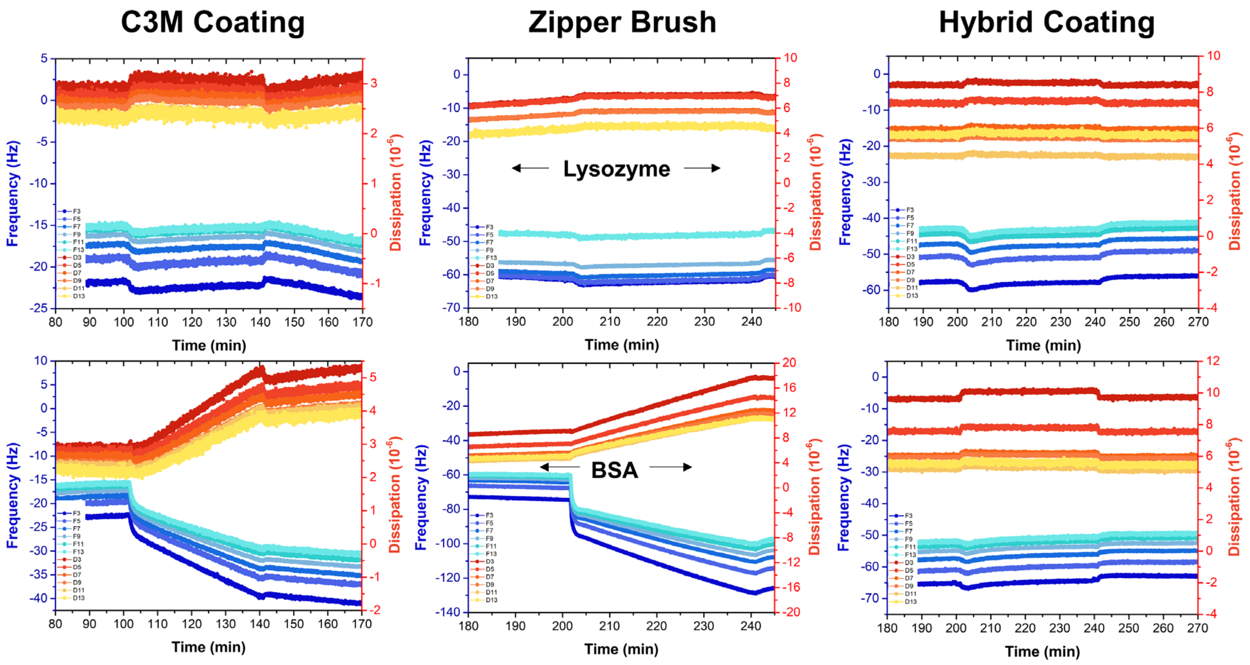

Figure S24. QCM-D graphs representing the antifouling performance of all three polymer-based coatings against lysozyme (top) and BSA (bottom). While they all successfully minimize the attachment of lysozyme, only the hybrid coating is able to effectively suppress the adhesion of both fouling agents, as is represented by its unaffected frequency signals.

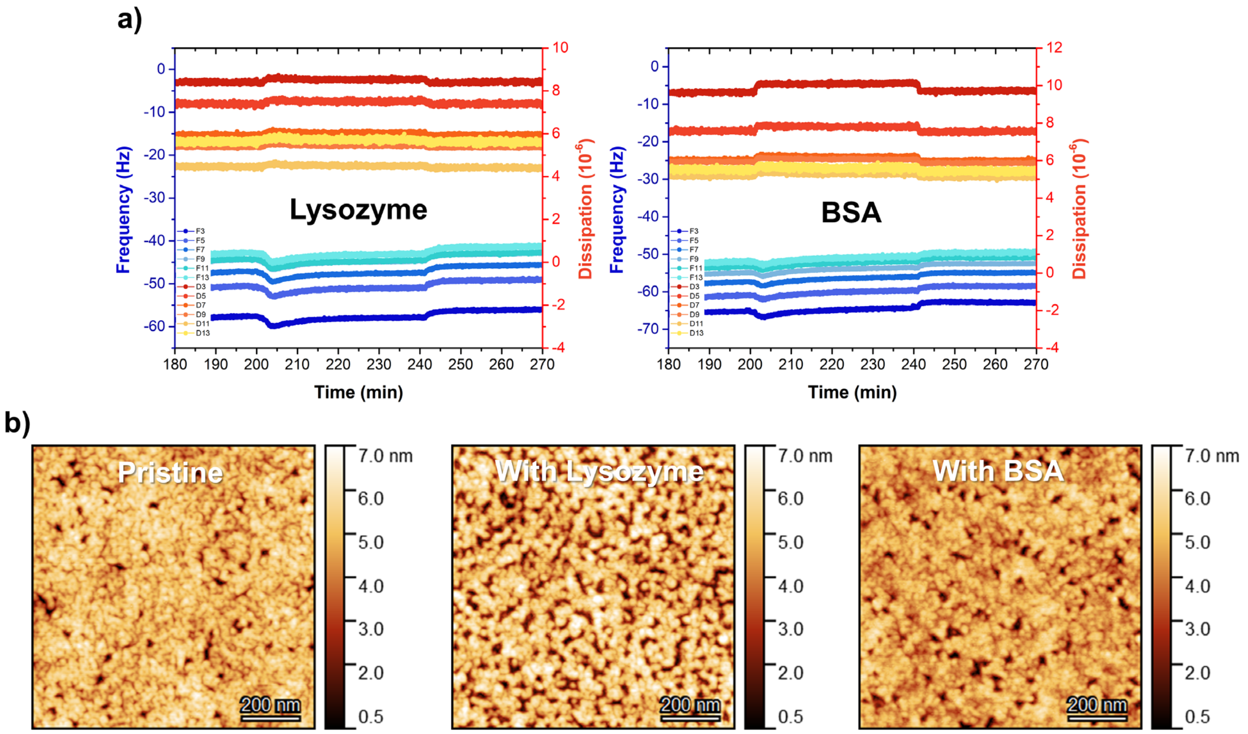

Figure S25. (a) The QCM-D graphs demonstrate the effective antifouling performance of the hybrid coating against both lysozyme and BSA. (b) The AFM height images further confirm poor adhesion of these fouling agents, as the final topography is barely affected after fouling testing.

Abbreviations: C3M, complex coacervate core micelle; PEG, poly(ethylene glycol); mPEG, methoxy poly(ethylene glycol); PU, polyurethane; PVDF, polyvinylidene fluoride; PEGMA, poly(ethylene glycol) methacrylate; VIPS, vapor-induced phase separation; PS, polystyrene; 1,2-PB, cross-linked 1,2-polybutadiene; PDMS, poly(dimethylsiloxane); PBS, phosphate-buffered saline (phosphate buffer); KNO_3, potassium nitrate salt.

Coating	Fabrication	Substrate	Adsorption Conditions	BSA Reduction	Lysozyme Reduction	Ref.
Hybrid Coating	Adsorption	PS	10 mM PBS pH = 8.0	> 99%	> 99%	This work
Zipper Brush	Langmuir-Blodgett + Adsorption	PS	10 mM KNO_3 pH = 6.0	98%	100%	[7]
C3M-PEG_{204}	Adsorption	PS	50 mM PBS pH = 7.7	26%	92%	[8]
C3M-PEG_{212} Coating	Adsorption	1,2-PB	10 mM PBS pH = 7.7	95%	66%	[9]
mPEG-PU Matrix	Molding	Free-standing	-	100%	< 0%	[10]
PVDF/PEGMA Matrix	VIPS	PVDF	-	93%	85%	[11]
PEG Monolayer	Grafting-to	PDMS	-	83%	81%	[12]

Table S2. Overview of previously reported PEG-based coatings fabricated on hydrophobic surfaces and their antifouling efficiency. The reduction of protein adsorption compared to the bare substrate is given in %.

In order to fairly assess and compare the antifouling efficiency of our hybrid coating to others, it was decided to only select antifouling coatings that satisfy the following criteria: the coating must be (1) PEG-based, (2) applied to a hydrophobic substrate and (3) tested against both BSA and lysozyme. Hence, antifouling coatings that involve other types of substrates, charged or zwitterionic antifouling groups and/or are only tested against either BSA or lysozyme, were not included.

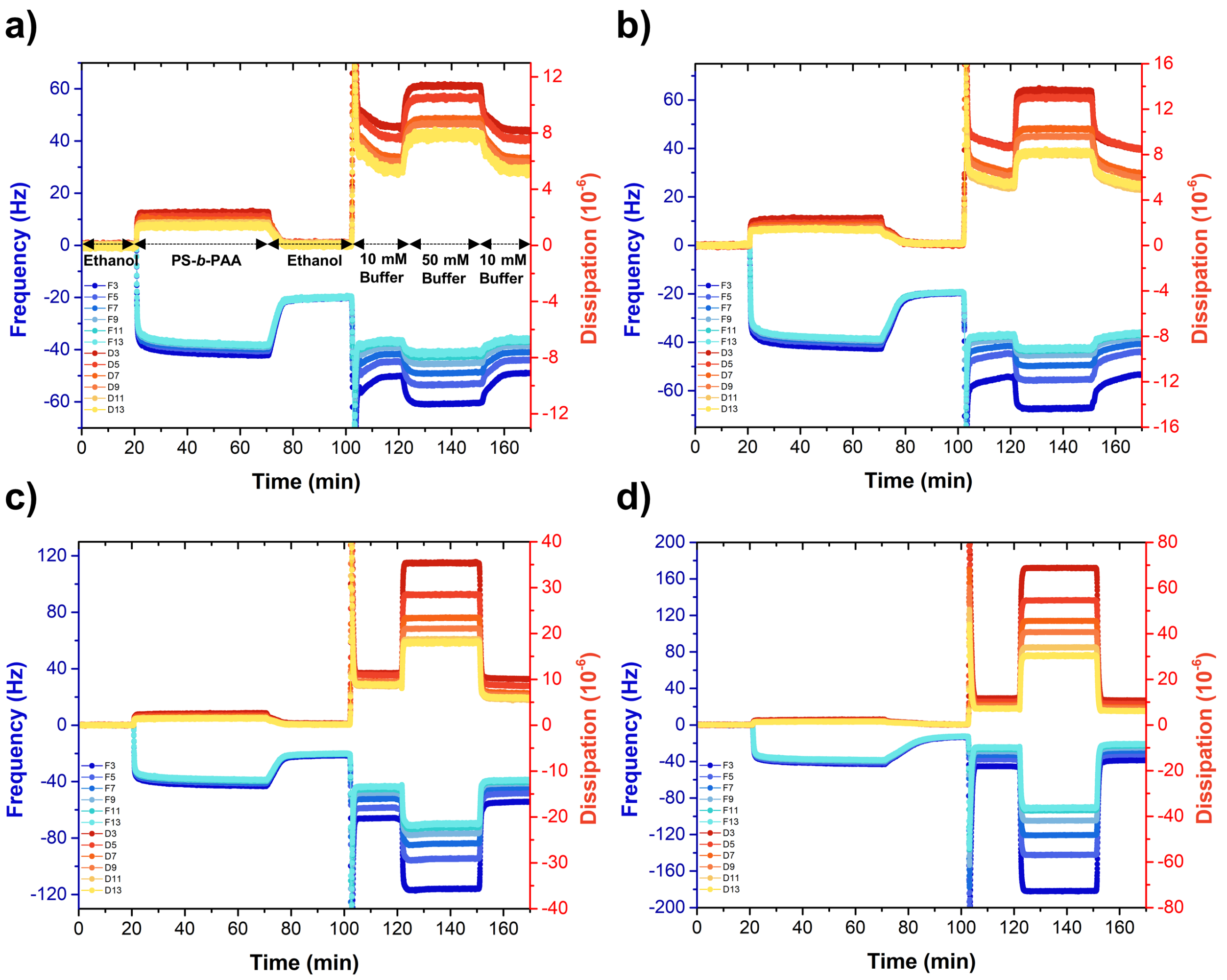

Figure S26. QCM-D data representing the in situ formation of PS-*b*-PAA primer (0 - 100 min), followed by a stability test against solutions of varying ionic strengths, including (a) 50 mM, (b) 100 mM, (c) 500 mM and (d) 1.0 M. According to the QCM-D data, the film remains stable up to the highest salt concentration of 1.0 M: the frequencies shift back (close) to the initial values seen prior to the salt treatment. The increased frequency and dissipation shifts when switching to higher salt concentrations can be explained by extensive swelling of the film.

**Ionic Strength**	**Thickness** (nm)	***Θ*** (°)
50 mM	2.4 (± 0.1)	62.2 (± 2.7)
100 mM	2.2 (± 0.1)	64.3 (± 0.7)
500 mM	2.1 (± 0.1)	66.2 (± 2.9)
1.0 M	3.1 (± 0.1)	69.2 (± 2.1)

Table S3. Summary of the dry thickness and contact angle (*Θ*) of the PS-*b*-PAA primer after treatment with aqueous buffer solutions of varying ionic strengths. The similarity in dry thicknesses and contact angles indicates that the films are not affected by salt concentrations up to 1.0 M. The slight variations in dry thickness and contact angle may be related to differences in salt uptake and swelling behavior. Each denoted value represents the mean ± the standard deviation calculated from at least three different spots on the coated sensors.

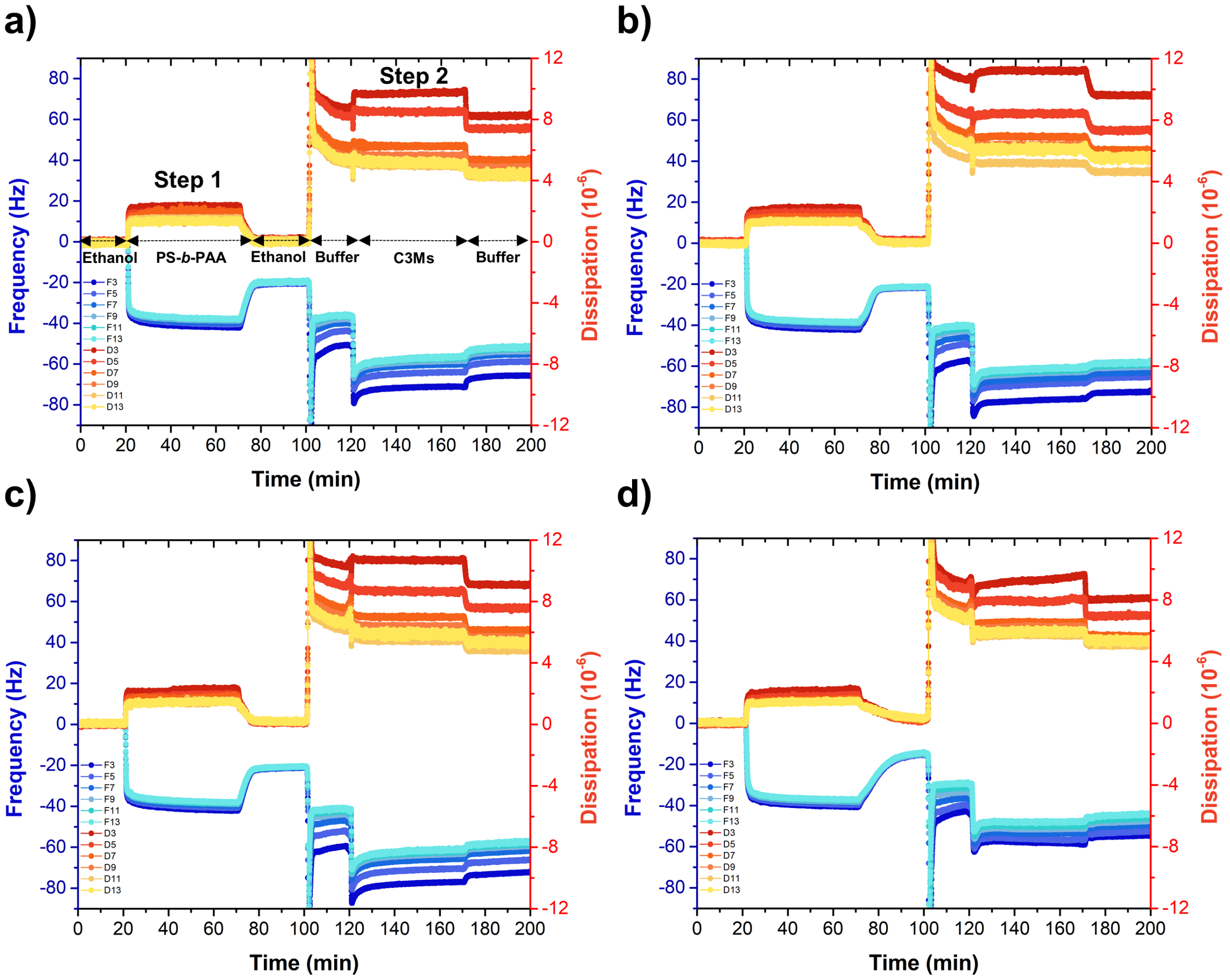

Figure S27. Four QCM-D graphs representing the highly reproducible two-step adsorption behavior of the hybrid coating, measured in parallel on four PS-coated sensors (a-d). The hybrid coating formation is characterized by two subsequent adsorption steps: adsorption of the PS-*b*-PAA micelles (step 1), followed by complexation to pre-fabricated C3Ms (step 2).

References

(1) Hofman, A. H.; Fokkink, R.; Kamperman, M. A Mild and Quantitative Route towards Well-Defined Strong Anionic/Hydrophobic Diblock Copolymers: Synthesis and Aqueous Self-Assembly. *Polym. Chem.* **2019**, *10* (45), 6109–6115. https://doi.org/10.1039/c9py01227c.

(2) Filippov, A. D.; Van Hees, I. A.; Fokkink, R.; Voets, I. K.; Kamperman, M. Rapid and Quantitative De-Tert-Butylation for Poly(Acrylic Acid) Block Copolymers and Influence on Relaxation of Thermoassociated Transient Networks. *Macromolecules* **2018**, *51* (20), 8316–8323. https://doi.org/10.1021/acs.macromol.8b01440.

(3) Obata, M.; Tanaka, S.; Mizukoshi, H.; Ishihara, E.; Takahashi, M.; Hirohara, S. RAFT Synthesis of Polystyrene-Block-Poly(Polyethylene Glycol Monomethyl Ether Acrylate) for Zinc Phthalocyanine-Loaded Polymeric Micelles as Photodynamic Therapy Photosensitizers. *J. Polym. Sci. Part A Polym. Chem.* **2018**, *56* (5), 560–570. https://doi.org/10.1002/pola.28929.

(4) Wu, T.; Gong, P.; Szleifer, I.; Vlček, P.; Šubr, V.; Genzer, J. Behavior of Surface-Anchored Poly(Acrylic Acid) Brushes with Grafting Density Gradients on Solid Substrates: 1. Experiment. *Macromolecules* **2007**, *40* (24), 8756–8764. https://doi.org/10.1021/ma0710176.

(5) Davis, K. A.; Matyjaszewski, K. Atom Transfer Radical Polymerization of Tert-Butyl Acrylate and Preparation of Block Copolymers. *Macromolecules* **2000**, *33* (11), 4039–4047. https://doi.org/10.1021/ma991826s.

(6) Pelras, T.; Hofman, A. H.; Germain, L. M. H.; Maan, A. M. C.; Loos, K.; Kamperman, M. Strong Anionic/Charge-Neutral Block Copolymers from Cu(0)-Mediated Reversible Deactivation Radical Polymerization. *Macromolecules* **2022**, *55* (19), 8795–8807. https://doi.org/10.1021/acs.macromol.2c01487.

(7) Vos, W. M. De; Kleijn, J. M.; Cohen Stuart, M. A. Polymer Brushes through Adsorption: From Early Attempts to the Ultra-Dense and Reversible "Zipper Brush." In *Polymer Brushes: Substrates, Technologies, and Properties*; 2012; pp 133–162.

(8) Brzozowska, A. M.; Hofs, B.; de Keizer, A.; Fokkink, R.; Cohen Stuart, M. A.; Norde, W. Reduction of Protein Adsorption on Silica and Polystyrene Surfaces Due to Coating with Complex Coacervate Core Micelles. *Colloids Surfaces A Physicochem. Eng. Asp.* **2009**, *347* (1–3), 146–155. https://doi.org/10.1016/j.colsurfa.2009.03.036.

(9) Hofs, B.; Brzozowska, A.; de Keizer, A.; Norde, W.; Cohen Stuart, M. A. Reduction of Protein Adsorption to a Solid Surface by a Coating Composed of Polymeric Micelles with a Glass-like Core. *J. Colloid Interface Sci.* **2008**, *325* (2), 309–315. https://doi.org/10.1016/j.jcis.2008.06.006.

(10) Golmohammadian Tehrani, A.; Makki, H.; Ghaffarian Anbaran, S. R.; Vakili, H.; Ghermezcheshme, H.; Zandi, N. Superior Anti-Biofouling Properties of MPEG-Modified Polyurethane Networks via Incorporation of a Hydrophobic Dangling Chain. *Prog. Org. Coatings* **2021**, *158*, 106358. https://doi.org/10.1016/j.porgcoat.2021.106358.

(11) Venault, A.; Ballad, M. R. B.; Huang, Y. T.; Liu, Y. H.; Kao, C. H.; Chang, Y. Antifouling PVDF Membrane Prepared by VIPS for Microalgae Harvesting. *Chem. Eng. Sci.* **2016**, *142*, 97–111. https://doi.org/10.1016/j.ces.2015.11.041.

(12) Guo, D. J.; Han, H. M.; Jing-Wang; Xiao, S. J.; Dai, Z. D. Surface-Hydrophilic and Protein-Resistant Silicone Elastomers Prepared by Hydrosilylation of Vinyl Poly(Ethylene Glycol) on Hydrosilanes-Poly(Dimethylsiloxane) Surfaces. *Colloids Surfaces A Physicochem. Eng. Asp.* **2007**, *308* (1–3), 129–135. https://doi.org/10.1016/j.colsurfa.2007.05.080.
